# Organoruthenium(II) Complexes Ameliorates Oxidative Stress and Impedes the Age Associated Deterioration in *Caenorhabditis elegans* through JNK-1/DAF-16 Signalling

**DOI:** 10.1038/s41598-018-25984-7

**Published:** 2018-05-16

**Authors:** G. Devagi, A. Mohankumar, G. Shanmugam, S. Nivitha, F. Dallemer, P. Kalaivani, P. Sundararaj, R. Prabhakaran

**Affiliations:** 1 Department of Chemistry, Bharathiar University, Coimbatore, 641 046 India; 2 Department of Zoology, Bharathiar University, Coimbatore, 641 046 India; 3 College of Science, Northeastern University, Boston, Massachusetts 02115 USA; 4 Laboratoire MADIREL CNRS UMR7246, Université of Aix-Marseille, Centre de Saint-Jérôme,bât. MADIREL, 13397 Marseille, Cedex 20 France; 5 Department of Chemistry, Nirmala College for Women, Bharathiar University, Coimbatore, 641018 India

## Abstract

New ruthenium(II) complexes were synthesised and characterized by various spectro analytical techniques. The structure of the complexes **3** and **4** has been confirmed by X-ray crystallography. The complexes were subjected to study their anti-oxidant profile and were exhibited significantly greater *in vitro* DPPH radical scavenging activity than vitamin C. We found that complexes **1–4** confered tolerance to oxidative stress and extend the mean lifespan of *mev-1* mutant worms and wild-type *Caenorhabditis elegans*. Further, mechanistic study and reporter gene expression analysis revealed that Ru(*ƞ*^6^*-p*-cymene) complexes maintained the intracellular redox status and offers stress resistance through activating JNK-1/DAF-16 signaling axis and possibly by other antioxidant response pathway. Notably, complex **3** and **4** ameliorates the polyQ (a Huntington’s disease associated protein) mediated proteotoxicity and related behavioural deficits in Huntington’s disease models of *C. elegans*. From these observations, we hope that new Ru(*ƞ*^6^*-p*-cymene) complexes could be further considered as a potential drug to retard aging and age-related neurodegenerative diseases.

## Introduction

The Huntington’s disease (HD) is an age associated, autosomal dominant, expansion of CAG trinucleotide repeat brain disorder characterized by loss of cognition, emotional turmoil and physical deterioration resulting in a progressive loss of neuronal structure and functions in striatum and cortex. In which, glutamine is encoded by CAG triplet within exon 1 of the huntingtin gene (*htt*), an ubiquitously expressed gene of anonymous function in all human beings. Mutation of the gene results in a polyglutamine tract (polyQ) near the N’ terminal of the mutant Htt (mHTT) which tend to aggregate. Several studies proved that the mechanisms of HD neuropathology are multifaceted in nature and it has been demonstrated that oxidative stress, neuronal excitotoxicity and mitochondrial dysfunction has been postulated as a key mechanisms consistently abnormal in HD experimental models as well as in post-mortem brains of HD patients^1–3^. Thus, we are in the need of novel therapeutic drug that could be used for the treatment of HD and associated pathogenesis. Decelerate the onset of disease progression by delaying the hallmarks of aging via therapeutic interventions are gaining increased attention from the scientific community in the recent decade. Considerable experimental evidences suggested that longevity promoting drugs are exceedingly useful in treatment of age associated diseases and several neurological conditions^4,5^.

The research for platinum based anticancer agents is the current interests, with particular attention to ruthenium^6,7^. Ru(II) arene complexes showed to have different profile of biological activity in comparison with the metal based anticancer complexes which are currently in clinical trials^8^. The ligand exchange kinetics of Pt(II) and Ru(II) complexes in aqueous solution, crucial for cytotoxicity, are very similar^9^. Furthermore, ruthenium compounds are found to be nontoxic and some of them were quite selective for cancer cells, likely due to the ability of ruthenium to mimic iron in binding to biomolecules^10^. Sadler *et al*., have found out the activity of neutral or cationic “half-sandwich” arene Ru(II) complexes^11,12^; these often possess good aqueous solubility and the arene ligand is somewhat inert towards displacement under physiological conditions^13,14^. Cationic ruthenium arene ethylenediamine complexes showed very high activity in both *in vitro* and *in vivo* studies^15–17^. Their interaction with DNA model compounds and further biologically related molecules has been established through simultaneous intercalation of extended aromatic groups, covalent coordination and stereospecific hydrogen bonding^18–21^. Arene ruthenium complexes with phosphine ligands, such as pta (1,3,5- triaza-7-phosphatricyclo[3.3.1.1]decane), as well as O,O- or N,O-chelating ligands, such as carboxylates, prevent hydrolysis, nevertheless this phenomenon does not reduces the cytotoxicity^22–25^. Though, limited efforts have been made in conjugating the metal centre with ligands that themselves show biological activity. Antitumor activity of Ru(II)arene compounds with curcumin, a well-known natural compound whose coordination chemistry has been only partially explored has been reported^26–31^. In addition, curcumin protects neurons against β-amyloid peptide toxicity and binds to β-amyloid plaques of transgenic mouse models of Alzheimer’s disease^32–34^. Thiosemicarbazones are a special class of chelating molecules with novel biological properties which make them as one of the potential candidates in exploring their activity when bind with the transition metals^35–40^. Binding of thiosemicarbazones with the transition metal ions drastically increases their pharmacological properties^41–43^.

Considering the advantageous features, we have chosen *Caenorhabditis elegans* (Nematoda, Rhabditidae) as an experimental model. Indeed, several signalling pathways and gerontogenes play a role in the signal transduction pathways associated with stress, longevity and proteostasis (protein homeostasis) in *C. elegans* are evolutionarily conserved. Among others, DAF-16 (the *C. elegans* fork head box O (FOXO) transcription factor) is the major transcription factor whose functions are highly facilitated by the insulin/IGF-1 (IIS) singling pathway. The reduced IIS signalling leads to the dephosphorylation and nuclear translocation of the DAF-16 transcription factor. Inside the nucleus, DAF-16 can either activates or represses the transcription of plethora of genes participated in cellular stress response, metabolism, autophagy, dauer formation, xenobiotic detoxification and protein homeostasis^44,45^. Furthermore, in our previous study, we found that organoruthenium(II) complexes promotes the DAF-16 nuclear accumulation and augments the expression downstream effector target SOD-3 in *C. elegans*^46^. Apart from the longevity promotion, DAF-16 also implicated with the progression of age related neurodegenerative disease including HD. A study showed that, DAF-16 and HSF-1 together reduced the polyQ aggregation via promoting the expression of small heat shock protein genes (sHSP), *hsp-12*.6, *hsp-1*6*.1*, *hsp-1*6*.49*, and *sip-1*^47^. The activation of DAF-16 can also directly assisted by the induction of stress related kinase JNK-1, the *C. elegans* homolog of c-Jun N-terminal kinase. In addition, over expression of JNK-1 stimulates the DAF-16 nuclear localization and regulates lifespan in a DAF-16 dependent manner^48,49^. JNK plays a pivotal role in a vast number of biological activities such as development and survival of cells in response to apoptosis and oxidative stress. A previous study reported that, JNK signalling appears to be required for normal lifespan and stress tolerance in *Drosophila melanogaster*^50^. In a recent work, it has been reported that 3β-Hydroxy-urs-12-en-28-oic acid extends the lifespan and prevents the polyQ aggregation *via* activation of JNK-1 and associated signalling in *C. elegans*^51^.

It was anticipated that the biological activity of the thiosemicarbazone moieties will be increased upon chelation with ruthenium and this idea made us to take up a detailed study on potential antioxidant and stress modulatory efficiency of the new ruthenium(II) *p*-cymene thiosemicarbazone complexes by using *C. elegans* as *in vivo* model. Also the molecular mechanism of complexes was examined using several mutant and transgenic forms of *C. elegans*. Furthermore, the potential beneficial effects of complexes on the onset and progression of HD, a protein misfolding disease were explored. Our results provide a great insight into the potential use of organoruthenium(II) complexes as an antioxidant and stress modulatory drug.

## Results and Discussion

**Figure 1:**
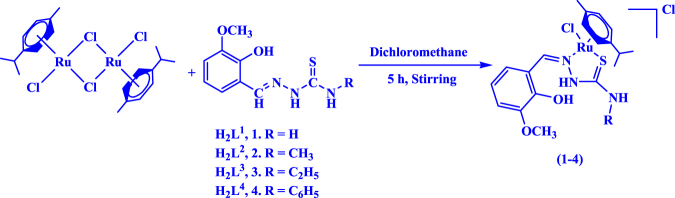
Synthesis of new ruthenium(II) complexes.

The reactions of 1:2 molar ratio of [(*ƞ*^6^-*p*-cymene)RuCl_2_]_2_ with various 3-methoxysalicylaldehyde-4(*N*)-substituted thiosemicarbazones (**H**_**2**_**L**^**1**^–**H**_**2**_**L**^**4**^) in dichloromethane resulted in the formation of four new complexes, the analytical data of which confirmed the stoichiometry of the complexes (**1–4**) (Fig. 1). The structure of the complexes **3** and **4** were confirmed by X-ray crystallographic studies and attempts were made to grow single crystals of complex **1** and **2** suitable for crystallographic studies in various organic solvents were unsuccessful. The complexes are found to be soluble in common organic solvents such as dichloromethane, chloroform, benzene, acetonitrile, ethanol, methanol, dimethylformamide, dimethylsulfoxide and H_2_O.

### Spectroscopic studies

The IR spectra of [**H**_**2**_**L**^**1**^–**H**_**2**_**L**^**4**^] showed a sharp band at 1539–1593 cm^−1^ corresponding to the ν_C = N_ vibration of azomethine group (Table 1**)** and this band has been observed at 1574–1593 cm^−1^ in the complexes, indicating the coordination of the azomethine nitrogen atom to the ruthenium^52^. A sharp band that appeared at 783–818 cm^−1^ in the ligands corresponding to ν_C=S_ vibration was appeared at 771–883 cm^−1^ in the complexes confirmed the binding of thione sulphur atom to the ruthenium^53^. Vibrations corresponding to phenolic OH (ν_OH_) was found at 3439–3457 cm^−1^ in the ligands shifted slightly lower region at 3427–3454 cm^−1^ in complexes (**1–4**), indicating the nonparticipation of the phenolic oxygen atom in coordination^54^. The electronic spectra of the complexes (**1–4**) (Supplementary Fig. S1) have been recorded in dichloromethane, and they displayed two to three bands in the region around 210–342 nm. The bands appearing in the region 210–291 nm have been assigned to intra ligand transitions^55^, and the band at 342 nm have been assigned to ligand to metal charge transfer transitions (LMCT)^56^. The ^1^H NMR spectral data of the ligands and the complexes were recorded in the DMSO and CDCl_3_. The ^1^H-NMR spectral data of the complexes in CDCl_3_ suggested a 1:1 molar ratio of the coordinated *p*-cymene and the Schiff base ligands (**H**_**2**_**L**^**1**^–**H**_**2**_**L**^**4**^). The proton NMR spectra and the detailed description have been given in supporting information (Supplementary Figs S2–S13). The molar conductance for complex **1** and **2** was found to be 122 Ω^−1^cm ^2^mol^−1^ and 113 Ω^−1^cm ^2^mol^−1^ respectively found to be in good agreement with the reported 1:1 electrolytic behaviour^57^.

**Table 1:** Crystallographic data of complexes [Ru(*ƞ*^6^-*p*-cymene)(MSal-etsc)Cl].Cl (**3**) and [Ru(*ƞ*^6^-*p*-cymene)(MSal-ptsc)Cl].Cl (**4**).

CCDC No.	[Ru(ƞ^6^-*p*-cymene)(MSal-etsc)Cl].Cl (3)	[Ru(ƞ^6^-*p*-cymene)(MSal-ptsc)Cl].Cl (4)
	1570571	1570572
Empirical formula	C_21_H_28_Cl_2_N_3_O_2_RuS	C_25_H_31_Cl_2_N_3_O_3_RuS
Formula weight	558.49	625.56
Temperature	293 K	293 K
Wavelength	0.71073 Å	1.54184 Å
Crystal system	monoclinic	Monoclinic
Space group	P2_1_/n	P 2_1_/c
a	9.9066(4) Å	13.39352(8) Å
b	14.3359(4) Å	11.20615(8) Å
c	17.9298(6) Å	18.31082(11) Å
α	90°	90°
β	92.228(4)°	97.7958(6)°
γ	90°	90°
Volume	2544.46(16)	2722.87(3)
Z	4	4
Calculated density	1.458 Mg/m^3^	1.526 Mg/m^3^
Absorption coefficient	0.929 mm^−1^	7.441 mm^−1^
F(000)	1140.0	1280.0
Crystal size	0.22 × 0.16 × 0.1 mm^3^	0.15 × 0.12 × 0.06 mm^3^
Theta range for data collection	3.326 to 28.548°	4.638 to 70.943°
Limiting indices	−13 ≤ h ≤ 12, −19 ≤ k ≤ 17, −23 ≤ l ≤ 23	−16 ≤ h ≤ 16, −13 ≤ k ≤ 12, −22 ≤ l ≤ 22
Reflections collected/unique	56198/5893 [R_int_ = 0.0662]	30138/5223 [R_int_ = 0.0214]
Completeness to theta	24.66 99.67%	64.99 99.96%
Absorption correction	Semi-empirical from equivalents	Semi-empirical from equivalents
Max. and min. transmission	1.00000 and 0.69503	1.00000 and 0.67976
Refinement method	Full-matrix least-squares on *F*^*2*^	Full-matrix least-squares on *F*^*2*^
Data/restraints/parameters	5893/4/282	5223/0/321
Goodness-of-fit on F^2^	1.058	1.042
Final R indices [I > 2sigma(I)]	R_1_ = 0.0615, wR_2_ = 0.1032	R_1_ = 0.0203, wR_2_ = 0.0524
R indices (all data)	R_1_ = 0.1058, wR_2_ = 0.1162	R_1_ = 0.0214, wR_2_ = 0.0531
Largest diff. peak and hole	0.69 and −0.77	0.67 and −0.45

### X-ray Crystallography

**Figure 2:**
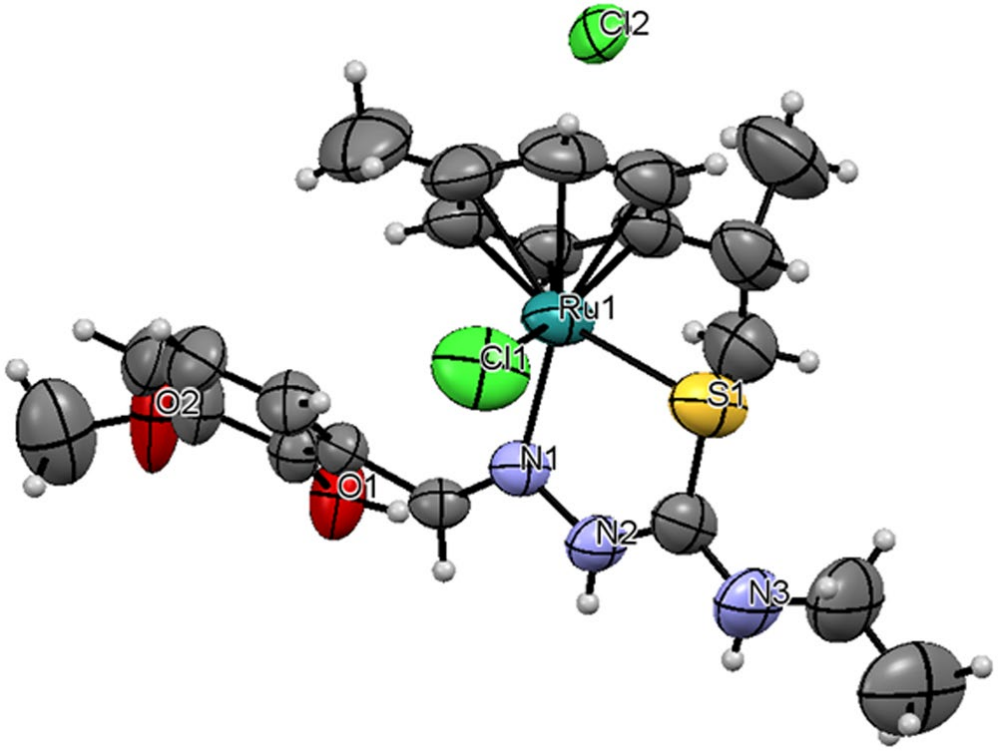
ORTEP for [Ru(*ƞ*^6^-*p*-cymene)(MSal-etsc)Cl].Cl (**3**) showing thermal ellipsoids at the 50% probability level.

**Figure 3:**
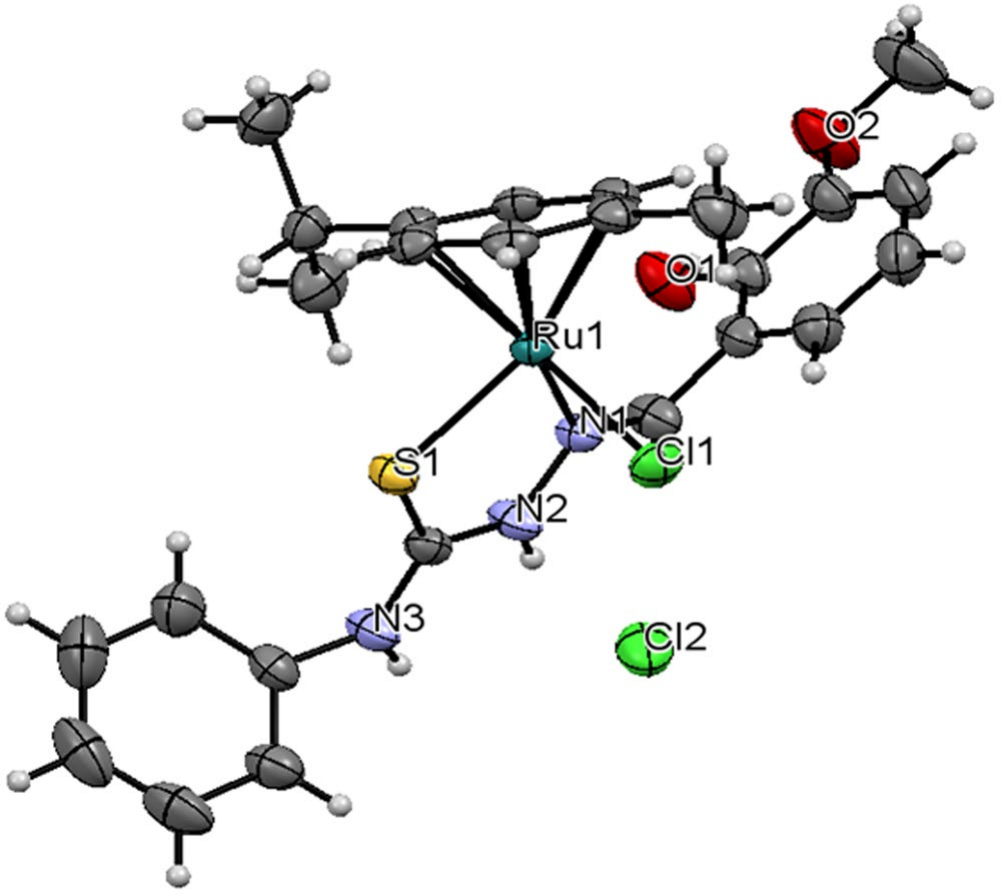
ORTEP for [Ru(*ƞ*^6^-*p*-cymene)(MSal-ptsc)Cl].Cl (**4**) showing thermal ellipsoids at the 50% probability level.

Complexes **3** and **4** crystallized in monoclinic space group P2_1_/n and P2_1_/c. The crystal structure of the complexes **3** and **4** are shown in Figs 2 and 3. Crystals of complexes **3** and **4** suitable for X-ray diffraction analysis were grown by slow diffusion of hexane into dichloromethane. Crystallographic data are listed in Table 1. Selected bond lengths and angles are listed for each compound in Supplementary Table S1. The molecular packing diagrams of the complexes were given in Supplementary Figs S14 and S15. In complex **3** and **4**, the ligand [H_2_-MSal-etsc] and [H_2_-MSal-ptsc] is coordinated to ruthenium ion through the N(1) nitrogen and thione sulfur atoms, forming a stable five-member chelate ring with a bite angle N(1)–Ru(1)–S(1) of 82.27(9)° and 82.06(4)° respectively. The Ru(1)–N(1) bond distance is 2.113(3) and 2.1200(14) Å, and the Ru(1)–S(1) distance is 2.3519(4) and 2.3519(4) Å for the complexes **3** and **4** respectively. The other two sites are occupied by chloride atom and *ƞ*^6^-*p*-cymene ring. The bond distance for Ru(1)−Cl(1) for **3** and **4** is 2.3898 (16) and 2.4055 (4) Å, respectively. The complexes are approximately octahedral with the *ƞ*^6^-*p*-cymene ring π-bonded to the ruthenium atom and occupying one face of the octahedron^55^. The other three sites are occupied by the chloride and the N,S donor Schiff base ligand. The ruthenium atom is situated 1.690 Å and 1.452 Å away from the centre of the *ƞ*^6^-*p*-cymene ring for complexes **3** and **4** respectively. The ruthenium–centroid distances are also in agreement with other structurally characterized cationic *ƞ*^6^-*p*-cymene complexes of ruthenium^58^. The Ru–S, Ru–Cl, and Ru–N distances are all in line with other structurally characterized *ƞ*^6^-arene ruthenium(II) complexes^58^. The variations in bond lengths and angles lead to a significant distortion from an ideal octahedral geometry for the complex^59^.

**Figure 4:**
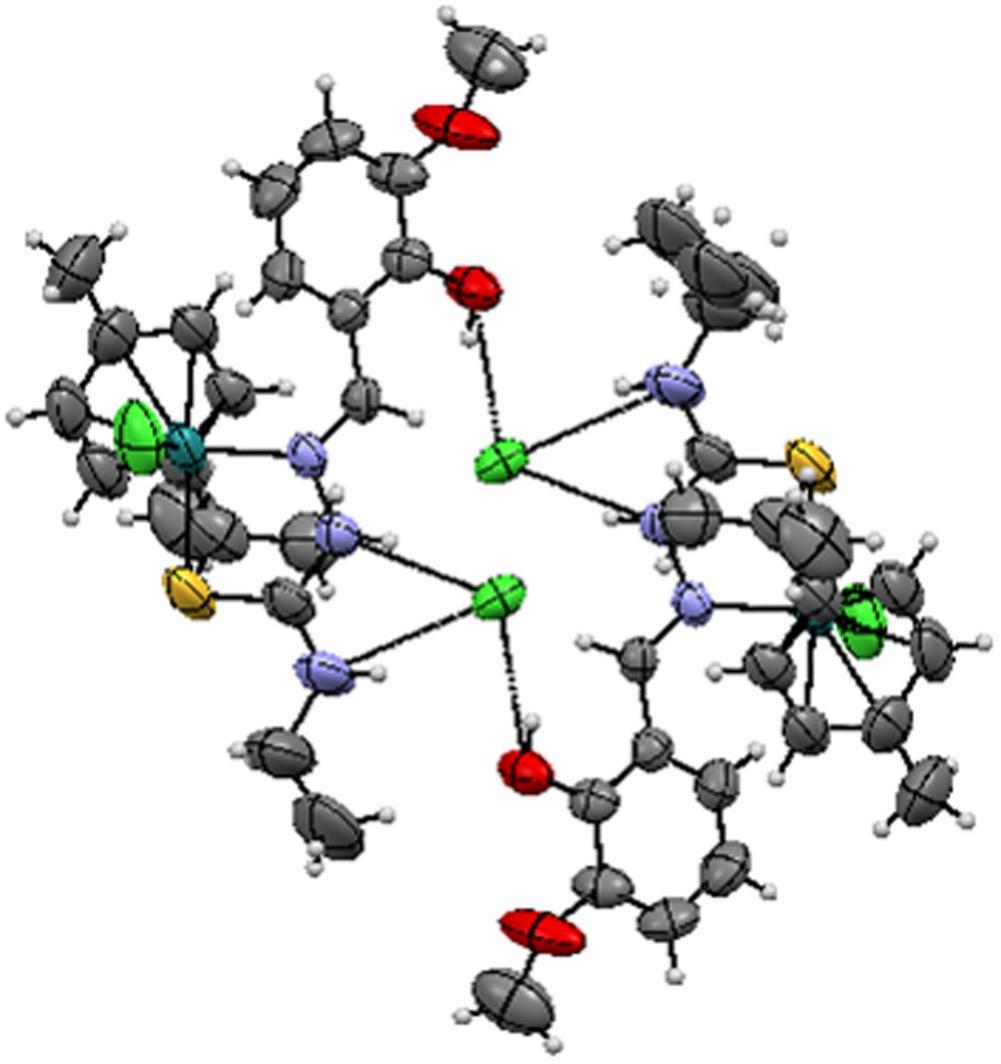
Hydrogen bonding of [Ru(*ƞ*^6^-*p*-cymene)(MSal-etsc)Cl].Cl **(3)**.

**Figure 5:**
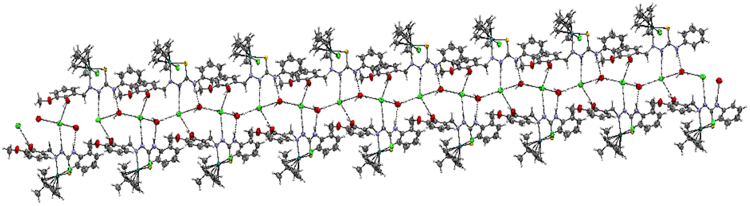
Hydrogen bonding of [Ru(*ƞ*^6^-*p*-cymene)(MSal-ptsc)Cl].Cl **(4)**.

In addition, complex **3** contains three intermolecular hydrogen bonding through the hydrogen atom of the hydroxy group with the chloride atom (Cl2), hydrogen atom of the hydrazinic nitrogen (N2) group with the chloride atom (Cl2) and hydrogen atom of the terminal nitrogen (N3) group with the chloride atom (Cl2) with O(1)–H(1)···Cl(2), N(2)–H(2)···Cl(2) and N(3)–H(3)···Cl(2) distance of 2.993 Å, 3.092 Å and 3.276 Å respectively. (Fig. 4, Supplementary Table S2), whereas complex **4** contains five intermolecular hydrogen bond through the hydrogen atom of the hydroxy group with the chloride atom (Cl2), hydrogen atom of the hydrazinic nitrogen (N2) group with the chloride atom (Cl2), hydrogen atom of the terminal nitrogen (N3) group with the chloride atom (Cl3) of another molecule, hydrogen atom (H3A) of water molecule with chloride atom (Cl2) and another hydrogen atom (H3B) of the same water molecule with another chloride atom (Cl2) with O(1)–H(1) ···Cl(2), N(2)—H(2)···Cl(2), N(3)—H(3)···O(3), O(3)—H(3A)···Cl(2), O(3)—H(3B)···Cl(2) distance of 3.036, 3.224, 2.763(3), 3.203(2) and 3.15(2) respectively, lead to the formation 2D double layer structure (Fig. 5).

### Ru(*ƞ*^6^*-p*-cymene) complexes exhibiting excellent *in vitro* and *in vivo* antioxidant activity

The antioxidant activity of biologically active compounds must be evaluated by several methods. Hence, to understand the possible mechanism, *in vitro* and *in vivo* experiments were carried out for evaluating the antioxidant activities of new Ru(*ƞ*^6^*-p*-cymene) complexes. Result depicted in Table 2 showed that all complexes exhibited significantly greater *in vitro* radical scavenging activity, as it was able to quench DPPH radicals stronger than vitamin C (V_C_). The radical scavenging activity of complexes **1**–**4** in the ranges of IC_50 DPPH_ = 0.211 ± 0.005, 0.274 ± 0.012, 0.192 ± 0.001 and 0.266 ± 0.013 μg/mL, respectively as compared to that of standard control V_C_ (5.139 ± 0.098 μg/mL). It is worth mentioning that ligands and starting precursor were demonstrated lower DPPH radical scavenging activity as compared to complexes and conventional standard. Considering the antioxidant potency, we then assayed the *in vivo* antioxidant efficiency using a nematode model *C. elegans*. In the *in vivo* assay system, we first assessed the safety property of Ru(*ƞ*^6^*-p*-cymene) complexes on wild type *C. elegans*. When assessing the safety property, we found that exposure with 0–18 µM concentration of the complexes **1**–**4** did not significantly alter the survival, development and reproduction of nematodes (Supplementary Fig. S16). In addition, ligands and precursor were found to be less toxic and affects the reproduction and development. In *C. elegans*, D-type GABAergic motor neurons regulate the locomotion behaviour^60^. It was found that complexes **1**–**4** did not obliviously alter the development/morphology of D-type GABAergic motor neurons. On the contrary, ligands and precursor exhibited some toxicity on the morphology of D-type GABAergic motor neurons (Supplementary Fig. S17). Neurons and reproductive organs are the two important secondary targets of any toxins and drugs in *C. elegans*. From these observations, it was apparent that new complexes are relatively safe in *C. elegans*. Hence, the ligands and precursor were excluded for the following experiments.

**Table 2:** *In vitro* free radical scavenging activities of ligands, starting precursor and complexes.

S. No	Particulars	Compounds	No. of Trials	IC_50_ (µg/mL) Mean ± SD
1.	Ligands	H_2_L^1^	2	439.88 ± 1.439
		H_2_L^2^	2	470.43 ± 11.469
		H_2_L^3^	2	288.86 ± 3.677
		H_2_L^4^	2	537.04 ± 6.300
2.	Precursor	[Ru(*p*-cymeme)Cl_2_]_2_	2	319.02 ± 9.472
3.	Complexes	**1**	3	0.266 ± 0.013
		**2**	3	0.274 ± 0.012
		**3**	3	0.192 ± 0.001
		**4**	3	0.211 ± 0.005
4.	Standard	Vitamin C	3	5.139 ± 0.098

All complexes showed far similar radical scavenging activity.

**Figure 6:**
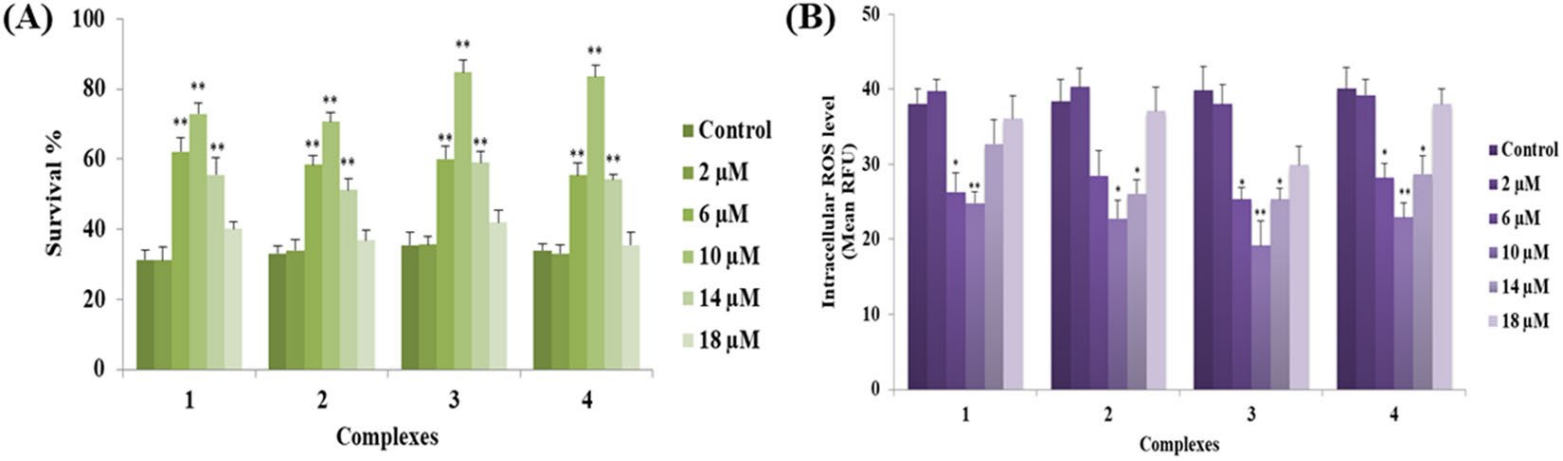
Effect of new Ru(*ƞ*^*6*^*-p*-cymene) on oxidative stress resistance and intracellular ROS levels in wild-type *C. elegans*. (**A**) Pretreatment of complexes **1–4** significantly (*P* < 0.001) promote the resistance to juglone-induced oxidative stress. (**B**) Quantification of intracellular ROS levels in N2 *C. elegans* using H_2_DCF-DA. The relative formation of juglone-induced intracellular ROS was reduced significantly (*P* < 0.05/*P* < 0.001) after treated with complexes **1–4**. Data are presented as mean ± SEM of three independent runs (n = 25–30/replicate), *P* values were calculated by Bonferroni post hoc test. *P < 0.05 and **P < 0.001.

**Figure 7:**
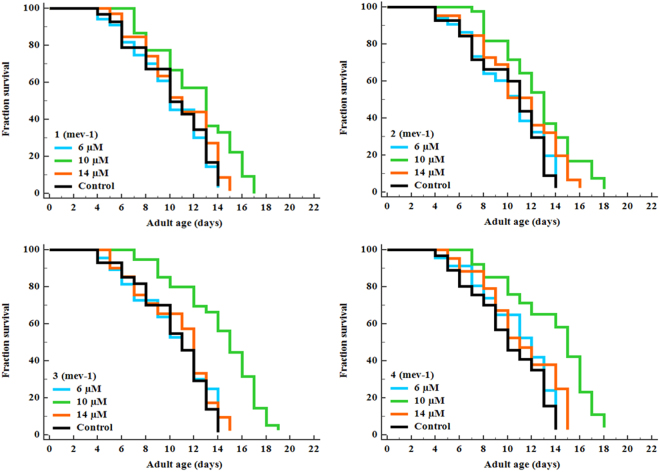
New Ru(*ƞ*^*6*^*-p*-cymene) complexes extend the lifespan of *mev-1* deficient *C. elegans*. Survival differences of *C. elegans* (n = 25–30/experiment) was estimated using Kaplan-Meier survival curves and analysed by the log-rank test using MedCalc software. See Supplementary Table S3 for statistical analysis and longevity data.

We have shown that pretreatment with complexes **1**–**4** at different pharmacological doses (2, 6, 10, 14 and 18 μM) significantly inhibited the induction of ROS generation and increased the survival rate of *C. elegans* under juglone intoxicated conditions (Fig. 6). We also performed lifespan assay using mitochondrial mutant strain TK22 [*mev-1* (*kn-1*)]. This strain has a mutation in *succinate dehydrogenase cytochrome b*, an integral membrane protein that is a subunit in complex 2 of mitochondrial respiratory chain. MEV-1 is required for oxidative phosphorylation, loss of function in *mev-1* resulted in the overproduction of free radicals and lead to a shortened lifespan^61^. We found that treatment with complexes **1**–**4** at 10 µM appeared to prolong the mean lifespan to 15.82%, 21.19%, 27.38% and 28.72% respectively (Fig. 7; Supplementary Table S3). The results indicated that the optimal dose (10 µM) of complexes **1**–**4** involved in the endogenous detoxification pathway thereby prolongs the lifespan of *mev-1* worms. Apart from metabolic control and gene expression pattern, the reduced level of ROS has been concomitantly associated with lengthening of organismal lifespan and healthspan^62,63^. In general, the progression of aging has been linked with declined redox regulation which makes the organism vulnerable to lethal diseases^64^. With such understanding from previous literatures, we examined the lifespan of wild type worms raised on the NGM plates in the presence or absence of complexes **1**–**4** (6, 10 and 14 μM). Of the three doses, animals raised on 10 μM of complexes **1**–**4** had increased the mean lifespan of N2 animals (p < 0.0001) to 12.60%, 16.64%, 22.32% and 22.95% respectively (Supplementary Fig. S18 and Supplementary Table S3). In all these experiments, we have noticed that complexes **1**–**4** displayed a hormetic-like concentration-dependent biphasic effects on *C. elegans* (Figs 6 and 7 and Supplementary Fig. S18). Several previous studies showed that stress hormesis mechanism extend the lifespan of *C. elegans* and confers neuroprotection in *Danio rerio*^65–67^. The reduction in the ROS level can be anticipated to be the major reason behind oxidative stress resistance and prolongevity in *C. elegans*. Taken collectively, these findings confirmed that complexes **1**–**4** reduced the intracellular ROS accumulation, conferred the resistance to stress and extended the lifespan of *mev-1* mutant as well as wild type worms by its potent antioxidative properties. Herein it is interesting to note that all the complexes exhibited better antioxidative than the standard vitamin C. Among the complexes, the activity varied based on their N-terminal substitution. Complex **3**, with more electron donating ethyl exhibited better activity than the all other complexes and they follow the order **3** > **4** > **1** > **2 > **Vitamin C. The current results were well supported by previous studies^62,63,68–71^. Based on these results, 10 µM complexes **1**–**4** were selected as an effective concentration for most subsequent experiments.

### Ru(*ƞ*^6^*-p*-cymene) complexes regulates the JNK-1/DAF-16 signalling axis in *C. elegans* to resist against stress

**Figure 8:**
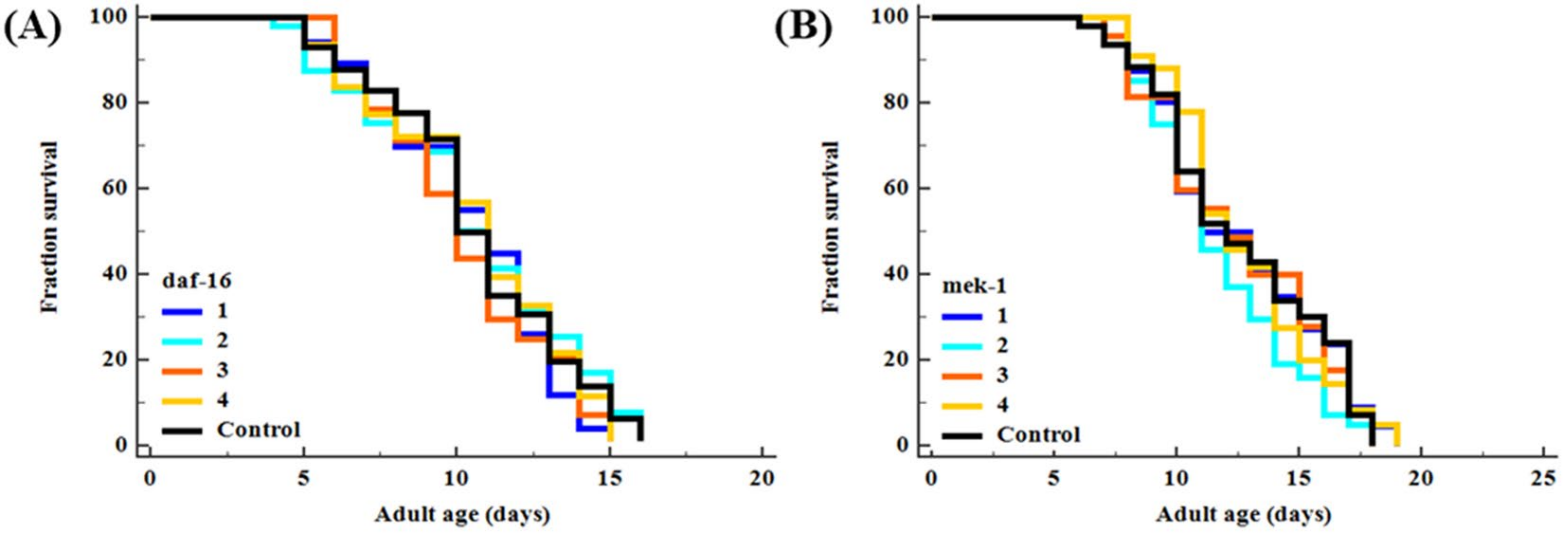
Stress modulatory and life-promoting effects of new Ru(*ƞ*^*6*^*-p*-cymene) complexes depend on JNK-1/DAF-16 signalling axis. Survival curve of (**A**) *daf-16* and (**B**) *mek-1* mutants treated with 10 µM of complexes **1–4**. Survival differences of *C. elegans* was estimated using Kaplan-Meier survival curves and analysed by the log-rank test using MedCalc software. The statistical details and longevity data were summarized in Supplementary Table S3.

**Figure 9:**
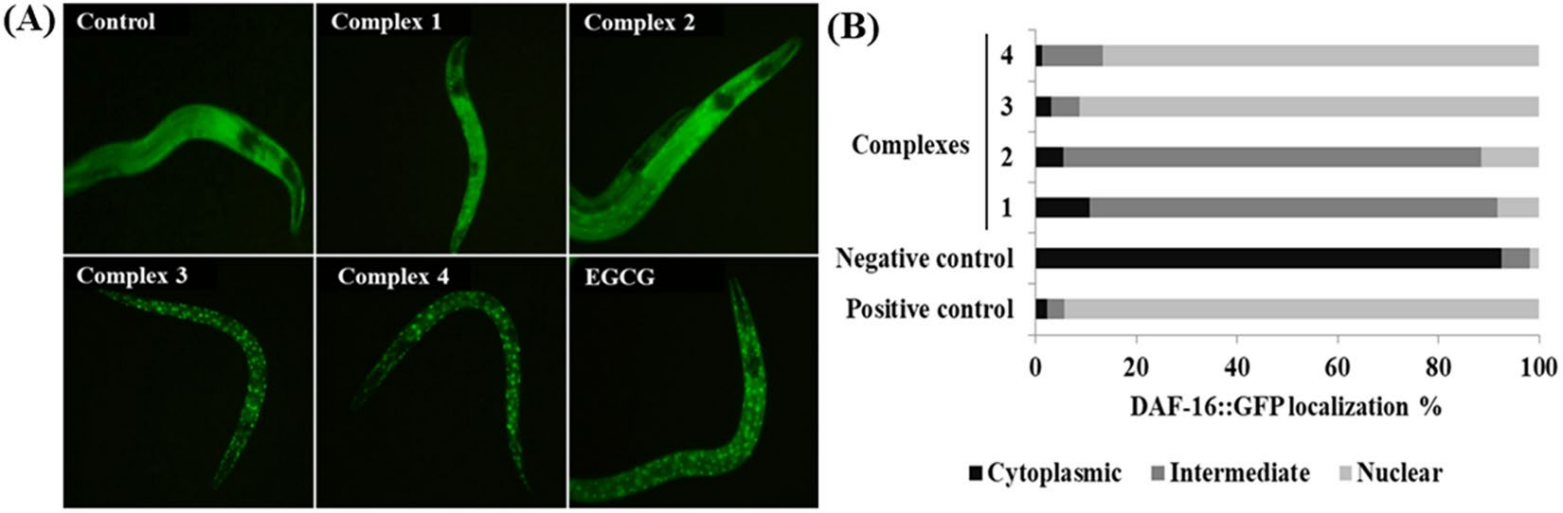
Ru(*ƞ*^*6*^*-p*-cymene) complexes activates the nuclear localization of DAF-16 in TJ356 *C. elegans*. (**A**) Fluorescence micrograph depicts the localization pattern of DAF-16. (**B**) Assessment of DAF-16 localization in control and complexes **1–4** treated *C. elegans*. EGCG and 0.1% DMSO was applied as positive and negative controls, respectively. Data are presented as mean ± SEM of three independent runs (n = 20–30/treatment). DAF-16 localization was classified into three groups namely cytoplasmic, intermediate and nuclear.

**Figure 10:**
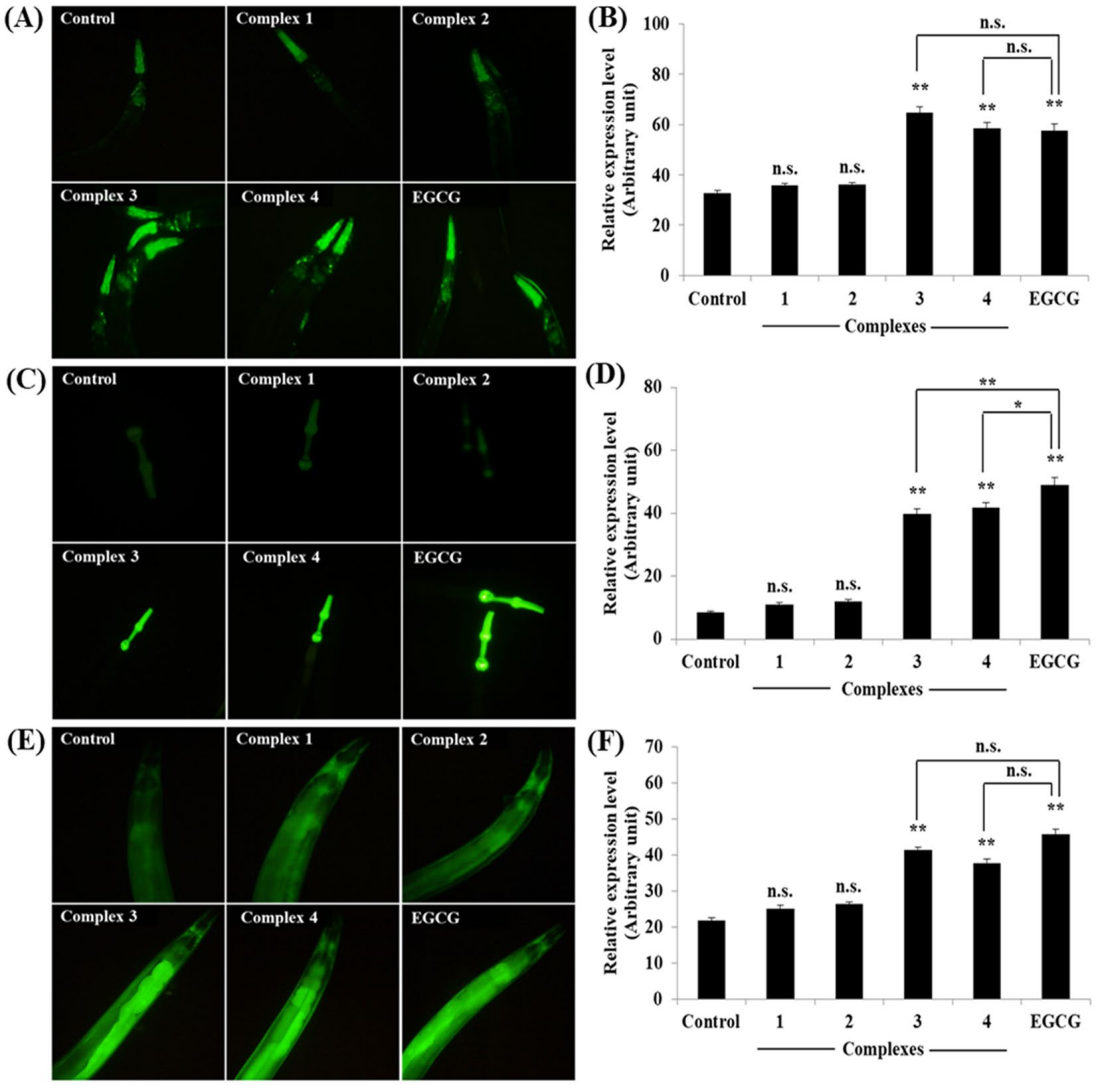
Ru(*ƞ*^*6*^*-p*-cymene) complexes induced the expression of stress-responsive genes in *C. elegans*. (**A**) *sod-3::GFP* expression, (**B**) quantification of *sod-3* expression, (**C**) *ctl-1,2,3::GFP* expression, (**D**) quantification of *ctl-1,2,3*, (**E**) *gst-4::GFP* expression, (**F**) quantification of *gst-4*. In all experiments was EGCG used as a positive control. Florescence intensity in pharynx for *sod-3::GFP* and *ctl-1,2,3:GFP* worms and full body for *gst-4::GFP* worms were quantified by image J and presented as relative expression rate (in arbitrary units). Representative bar graphs depicts the relative expression level of transgenes with SEM of three independent experiments (n = 20~30 individuals/experiment). *P* values were calculated by Bonferroni post hoc test, ^n.s.^not significant, *P < 0.05 and **P < 0.001.

To elucidate the molecular mechanism of stress resistance and life promoting effects of complexes **1–4**, we perused the lifespan assay using *daf-1*6 and *mek-1* mutant alleles. The DAF-16/FOXO is an evolutionary conserved transcription factor plays an indispensable role in the regulation of signal transduction pathways associated with stress modulation and longevity phenotype in *C. elegans*^72^. Prior genetic study has been probed that JNK-1, a member of mitogen-activated protein kinase (MAPK) family, promotes the lifespan of *C. elegans* in response to stress via DAF-16^73^. JNK-1 is positively regulated by the upstream MAP kinase kinase (MAPKK) super family protein MEK-1, which is encoded by *mek-1* gene. Our mechanistic study showed that, complexes **1**–**4** treatment fails to considerably increase the lifespan of *daf-16* and *mek-1* null mutants (Fig. 8; Supplementary Table S3). Moreover, all complexes failed to increase the survival rate of *daf-16* and *mek-1* mutant worms under juglone exposure, in contrast to results obtained with N2 *C. elegans* (Supplementary Fig. S19). The hormetically-induced life extension was further blocked by the mutation in *daf-16*^66,74^. It represents that DAF-16 is an essential transcription factor driving the hormesis-induced beneficial effects in *C. elegans* and it was consistent with previous results (i.e. lifespan and oxidative stress resistance of wild-type worms). Therefore, stress modulatory effects of complexes **1**–**4** is probably mediated through JNK-1/DAF-16 pathway^73^. To further confirm these results, we investigated whether complexes **1**–**4** treatment activates DAF-16 subcellular localization under normal condition. The complexes **1** and **2** treatments resulted in the partial/intermediate translocation of DAF-16, whereas a greater percentage of worms treated with **3** and **4** exhibited a nuclear relocation pattern of DAF-16 compared with that of untreated control group (Fig. 9). We then treated the transgenic worms, which contains *sod-3*::GFP, *hsp-16*.2::GFP *gst-4*::GFP and *ctl-1,2,3::GFP* reporter transgene with complexes **1–4**. As a result, complex **3** and **4** treated groups showed a significant increase of fluorescence intensity in CF1553, CL2070, CL2166 and GA800 *C. elegans* respectively. However, no corresponding increase in expressions were observed in the groups treated with complexes **1** and **2** (Fig. 10; Supplementary Fig. S20). All these genes offered a conserved protection against stress and its expressions can be modulated by antioxidants. Moreover, the master regulator of stress resistance and longevity, DAF-16 transcription factor, can also regulates the expression of these antioxidant gene (i.e. *sod-3*, *hsp-1*6*.2* and *ctl-1,2,3*)^62,75–78^. Thus, we concluded that, Ru(*ƞ*^6^*-p*-cymene) complexes, especially **3** and **4**, offers stress resistance not only by its redoubtable antioxidative potential but additionally by modulating the expression of stress-responsive genes in *C. elegans*.

### Ru(*ƞ*6*-p*-cymene) complexes alleviates PolyQ-mediated toxicity

**Figure 11:**
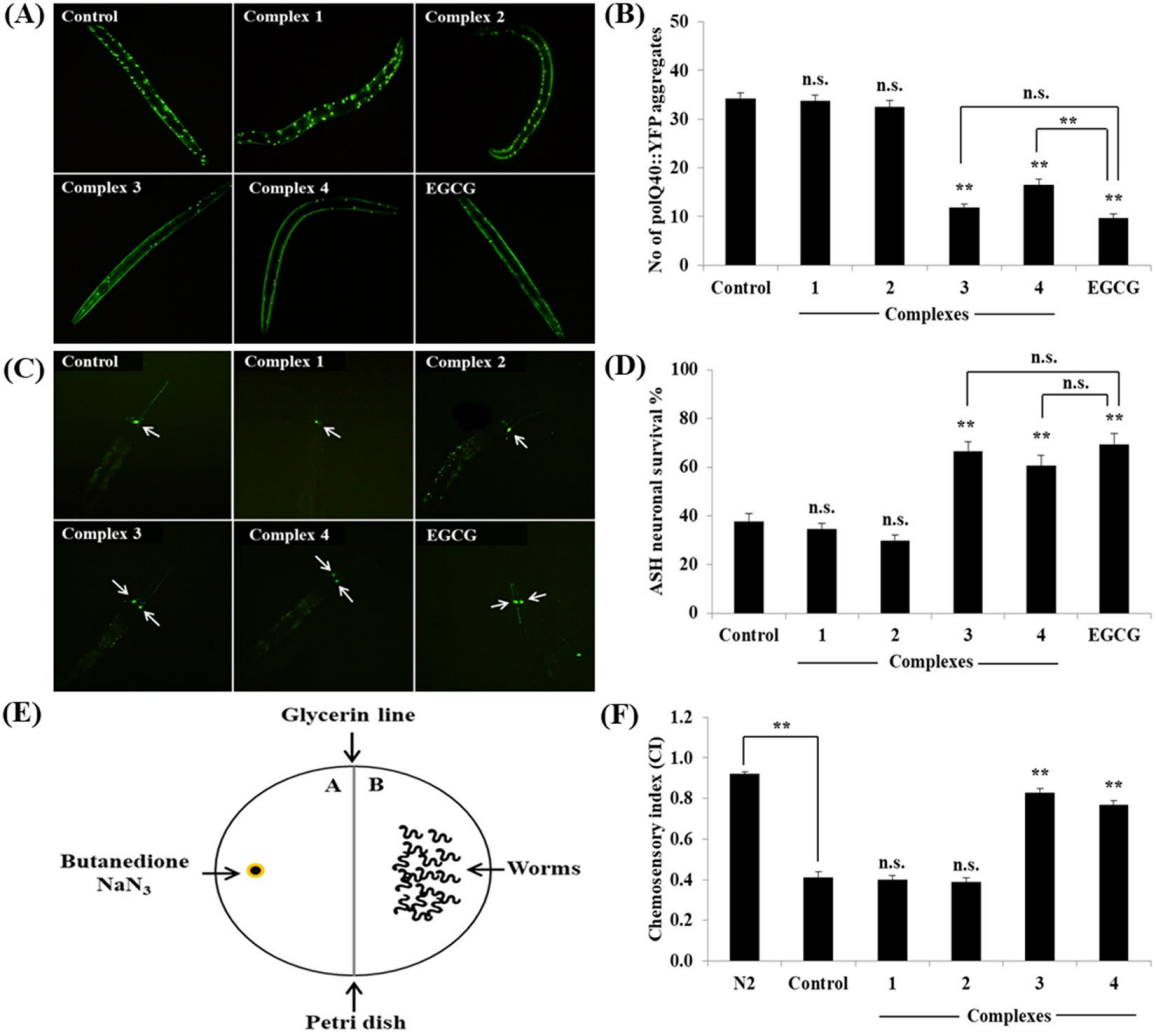
The new Ru(*ƞ*_*6*_*-p*-cymene) complexes confers the polyQ-mediated proteotoxicity and other HD related functional deficits in *C. elegans*. (**A**) Complexes **3** and **4** significantly (*P* < 0.001) reduced the polyQ40::YFP aggregates in AM141 *C. elegans*. (**B**) Quantification of polyQ40::YFP aggregation. (**C**) Complexes **3** and **4** protect the polyQ-mediated death of ASH neurons. (**D**) Quantification of ASH neuronal survival. (**E**) Schematic overview for chemosensory behaviour assay. (**F**) Effects of complexes on chemosensory behaviour of HA759 *C. elegans*. Bar graphs are expressed as mean ± SEM of three independent experiments (n = 20~30 individuals/replicate). *P* values were calculated by Bonferroni post hoc test, ^n.s.^not significant and **P < 0.001.

We also investigated the possibility of whether complexes **1**–**4** could reduce the Huntington’s disease (HD) associated pathologies in *C. elegans*. Results showed that complex **3** and **4** significantly reduced the polyQ (polyQ40::YFP) aggregate score, whereas complex **1** and **2** had no effects on polyQ aggregation in AM141 *C. elegans* (Fig. 11A). We next examined the protective effect of complexes against polyQ mediated neuronal death in HA759 *C. elegans* expressing polyQ tract (Htn-Q150::GFP) exclusively in ASH neurons. As shown in Fig. 11B, only 37.78 ± 3.02% ASH neurons were survived in control group worms which indicated that aggregation of polyQ induces the neuronal death. We did not observed any significant changes in the protection upon treatment with complex **1** and **2**, while complex **3** and **4** were proficient of increasing the neuronal survival at 10 μM to 66.67 ± 3.82% and 60.56 ± 4.37% respectively. At this molarity, complex **3** and **4** treatments found to be more effective in improving the chemosensory index to ~0.83 and ~0.77 respectively indicating the healthy status of ASH chemosensory neurons when compared to untreated (~0.41) and complex **1** (~0.40) - **2** (~0.39) treated groups (Fig. 11C). Therefore, this result suggests that complex **3** and **4** improved the intracellular protein homeostasis (proteostasis) via inhibition of unwanted proteomic changes in *C. elegans*. The impaired proteostasis network and accumulation of non-native protein aggregates in various tissues are common features of aging and neurodegenerative diseases including HD^79^. Emerging scientific evidences suggest that delaying the aging process and clearing the affected protein or preventing the aggregate formation might be an effective strategy to normalize the protein misfolding diseases^80–82^. These polyQ protein aggregations were greatly delayed or even halted by the influences of JNK-1^51^ and DAF-16^83^. A genetic study revealed that DAF-16 interrupt the formation of polyQ aggregation through the regulation of four small heat-shock factors (sHsp) genes *viz*., *hsp-1*6*.1*, *hsp-1*6*.49*, *hsp-12*.6 and *sip-1*, since these sHSP has had the DNA binding site for DAF-16^47^. In addition polysaccharide from *Astragalus membranaceus* offers protection against polyQ proteotoxicity through regulating DAF-16/FOXO pathway^84^. In the present study, we confirmed that Ru(*ƞ*^*6*^*-p*-cymene) complexes mediate stress resistance in *C. elegans* probably via JNK-1/DAF-16 pathway. Taken together, these results suggest that complexes exert anti-aggregative and neuroprotective activity through regulating stress-response pathways.

## Conclusion

The current work deals with the synthesis and characterization (analytical, IR, UV-Vis and NMR) of the new Ru(II)*p*-cymene complexes (**1–4**), which are obtained from the reaction between [RuCl_2_(η^6^-*p*-cymene)]_2_ and **H**_**2**_**L**^**1**^–**H**_**2**_**L**^**4**^. The structures of representative complexes **3** and **4** were confirmed by single-crystal X-ray diffraction techniques. *In vivo* results demonstrated that hormetic-like effect of new Ru(*ƞ*^*6*^*-p*-cymene) complexes extend the lifespan and mitigates the impact of exogenous stress through activating conserved JNK-1/DAF-16 signalling axis. In addition, complexes **3** and **4** remarkably protects the *C. elegans* from proteotoxic stress induced by polyQ, a HD associated toxic protein. Hence, further studies on higher models are required to elucidate the detailed mechanism of action of Ru(*ƞ*^*6*^*-p*-cymene) complexes before making into a drug candidate.

### Measurements

All the reagents used were analar grade, were purified and dried according to the standard procedure^85^. 3-methoxysalicylaldehyde, thiosemicarbazide, 4(*N*)-substituted thiosemicarbazides were obtained from Himedia. Melting points were measured in a Lab India apparatus. Infrared spectra were measured as KBr pellets on a Jasco FT-IR 400–4100 cm^−1^ range. Elemental analyses of carbon, hydrogen, nitrogen and sulphur were determined by using Vario EL III CHNS at the Department of Chemistry, Bharathiar University, Coimbatore, India. Electronic absorption spectra of the compounds were recorded in dichloromethane using JASCO 600 spectrophotometer. Molar conductance of the complexes was determined in dichloromethane at room temperature by using a Jenway model 4070 conductivity meter. ^1^H and ^13^C spectra were recorded in CDCl_3_ and DMSO at room temperature with a Bruker 400 MHz instrument, chemical shift relative to tetramethylsilane. The chemical shifts are indicated in parts per million (ppm). Single crystal data collections and corrections for the new Ru(II) complexes **3** and**4** were done at 293 K with CCD Kappa Diffractometer using graphite mono chromated Mo Kα (k = 0.71073 Å and 1.54184 Å) radiation^86^. The structure solutions were done by using SHELXS-97^87^ and refined by full matrix least square on *F*^2^ using SHELXL-2014^88^.

### Experimental Section

The ligand **[H**_**2**_**L**^**1**^–**H**_**2**_**L**^**4**^**]** and the ruthenium complex **[(ƞ**^**6**^**-*p*-cymene)RuCl**_**2**_**]**_**2**_ were synthesized according to the standard literature procedures^89–91^.

### Synthesis of new Ruthenium(II) complexes

#### Synthesis of [Ru(*ƞ*^6^-*p*-cymene)(MSal-tsc)Cl].Cl

To a solution of [(*ƞ*^6^-*p*-cymene)RuCl_2_]_2_ (0.05 g, 0.0816 mmol) in dichloromethane (10 cm^3^), 3-methoxysalicylaldehyde thiosemicarbazone [H_2_-Msal-tsc] (0.036 g, 0.1632 mmol) in dichloromethane (10 cm^3^) was added. The reaction mixture was then stirred at room temperature for 5 h. The reddish orange suspension gradually turned orange color. The solvent was removed under reduced pressure. The complex was recrystallized from dissolved in CH_2_Cl_2_/hexane. Yield: 83%. Mp 168 °C. Anal. calcd for C_19_H_25_Cl_2_N_3_O_2_RuS: C, 42.94; H, 4.74; N, 7.91; S, 6.03. Found: C, 42.93; H, 4.75; N, 7.89; S, 5.99%. FT-IR (cm^−1^) in KBr: 3363 (ʋ_O-H_), 1591 (ʋ_C = N_), 878 (ʋ_C=S_); UV-Vis (CH_2_Cl_2_), λ_max_: 210 (30,442), 226 (27,342) and 270 (28,354) nm (dm^3^ mol^−1^cm^−1^) (intra-ligand transition); ^1^H NMR (CDCl_3_, ppm): 10.1 (s, 1 H, –OH), 8.29 (s, 1 H, –CH = N), 6.99 (s, terminal –NH_2_), 3.90 (s, 3 H, –OCH_3_), 9.18 (s, 1 H, –NH-C = S), 7.35–7.45 (m, aromatic), 5.45–5.46 (d, J = 4.0 Hz, 1 H, Hcymene), 5.67–5.69 (d, J = 8.0 Hz, 1 H, Hcymene), 5.18–5.20 (d, J = 8.0 Hz, 1 H, Hcymene), 4.98–4.99 (d, J = 4.0 Hz, 1 H, Hcymene), 2.81–2.88 (pent, J = 6.8 Hz, 1 H, CH(Me)_2_), 1.86 (s, 3 H, CH_3_-cymene), 1.09–1.11 (d, J = 8.0 Hz, 3 H, CH(CH_3_)_2_). ^13^C NMR (CDCl_3_, ppm): δ 19.5 (CH_3_ of *p*-cymene), δ 21.5 (2CH_3_ of *p*-cymene), δ 31.8 (CH of *p*-cymene), δ 83.1–86.9 (aromatic carbons of *p*-cymene), δ 98.3 and δ 104.4 (quaternary carbons of *p*-cymene), δ 128.4, δ 130.7, δ 132.1, δ 133.3, δ 134.5, δ 137.5 (aromatic carbons), δ 178.1 (C = S), δ 160.5 (C = N), δ 147.9 (C-O), δ 40.6 (OCH_3_).

A similar method was followed to synthesize other complexes.

#### Synthesis of [Ru(*ƞ*^6^-*p*-cymene)(MSal-mtsc).Cl]Cl

Complex **2** was prepared by the procedure as described for (**1**) with 3-methoxysalicylaldehyde-4(N)-methylthiosemicarbazone [H_2_-Msal-mtsc] (0.036 g, 0.1632 mmol). The reddish orange suspension gradually turned orange color. The solvent was removed under reduced pressure. The complex was recrystallized from dissolved in CH_2_Cl_2_/hexane. Yield: 85%. Mp: 178 °C. Anal. calcd. for C_20_H_27_Cl_2_N_3_O_2_RuS: C, 44.04; H, 4.99; N, 7.70; S, 5.88. Found: C, 43.99; H, 4.80; N, 7.74; S, 5.92%. FT-IR (cm^−1^) in KBr: 3429 (ʋ_O-H_), 1597 (ʋ_C = N_), 869 (ʋ_C=S_); UV-Vis (CH_2_Cl_2_), λ_max_: 231 (50,054), 248 (70,772) and 291 (55,367) nm (dm^3^ mol^−1^ cm^−1^) (intra-ligand transition); ^1^H NMR (CDCl_3_, ppm): 14.58 (s, 1 H, –OH), 8.84 (s, 1 H, –CH = N), 8.03 (m, terminal –NH), 3.15 (s, 3 H, –OCH_3_), 8.99 (br s, 1 H, –NH-C = S), 2.08 (s, terminal –CH_3_), 7.00–7.06 (m, aromatic), 5.40–5.42 (d, J = 8.0 Hz, 1 H, Hcymene), 5.07–5.09 (d, J = 8.0 Hz, 1 H, Hcymene), 4.82–4.83 (d, J = 4.0 Hz, 1 H, Hcymene), 4.61–4.63 (d, J = 8.0 Hz, 1 H, Hcymene), 2.65–2.72 (pent, J = 6.8 Hz, 1 H, CH(Me)_2_), 1.59 (s, 3 H, CH_3_-cymene), 1.12–1.13 (d, J = 4.0 Hz, 3 H, CH(CH_3_)_2_), 1.16–1.18 (d, J = 8.0 Hz, 3 H, CH(CH_3_)_2_). ^13^C NMR (CDCl_3_, ppm): δ 18.5 (CH_3_ of *p*-cymene), δ 23.1 (2CH_3_ of *p*-cymene), δ 32.7 (CH of *p*-cymene), δ 83.3–87.9 (aromatic carbons of *p*-cymene), δ 100.9 and δ 104.2 (quaternary carbons of *p*-cymene), δ 127.7, δ 129.1, δ 131.2, δ 132.9, δ 133.9, δ 135.4 (aromatic carbons), δ 177.6 (C = S), δ 163.4 (C = N), δ 149.7 (C-O), δ 42.9 (OCH_3_), δ 37.83 (NH-CH_3_).

#### Synthesis of [Ru(*ƞ*^6^-*p*-cymene)(MSal-etsc)Cl].Cl

Complex **3** was prepared by the procedure as described for (**1**) with 3-methoxysalicylaldehyde-4(N)-ethylthiosemicarbazone [H_2_-Msal-etsc] (0.041 g, 0.1632 mmol). The reddish orange suspension gradually turned orange color. The solvent was removed under reduced pressure. The complex was recrystallized from dissolved in CH_2_Cl_2_/hexane. The orange crystals were obtained. Yield: 82%. Mp. 170 °C. Anal. Calcd for C_21_H_28_Cl_2_N_3_O_2_RuS: C, 45.08; H, 5.22; N, 7.51; S, 5.73. Found: C, 45.00; H, 5.18; N, 7.54; S, 5.80%. FT-IR (cm^−1^) in KBr: 3433 (ʋ_O-H_), 1589 (ʋ_C = N_), 883 (ʋ_C=S_); UV-Vis (CH_2_Cl_2_), λ_max_: 258 (132854) nm (dm^3^ mol^−1^cm^−1^) (intra-ligand transition); 342 (62,976) nm (dm^3^ mol^−1^cm^−1^) (LMCT); ^1^H NMR (CDCl_3_, ppm): 14.51 (s, 1 H, –OH), 8.85 (s, 1 H, –CH = N), 8.00–8.024 (d, J = 8.0 Hz, terminal –NH), 3.97 (s, 3 H, –OCH_3_), 9.02 (s, 1 H, –NH-C = S), 3.58–3.59 (d, J = 4.0 Hz, terminal –CH_2_), 1.30–1.34 (t, J = 7.2 Hz, terminal –CH_3_), 6.49–7.26 (m, aromatic), 5.404–5.418 (d, J = 5.6 Hz, 1 H, Hcymene), 5.070–5.085 (d, J = 8.0 Hz, 1 H, Hcymene), 4.819–4.831 (d, J = 6.0 Hz, 1 H, Hcymene), 4.60–4.62 (d, J = 8.0 Hz, 1 H, Hcymene), 2.59–2.72 (pent, J = 7.2 Hz, 1 H, CH(Me)_2_), 1.25 (s, 3 H, CH_3_-cymene), 1.12–1.14 (d, J = 8.0 Hz, 3 H, CH(CH_3_)_2_), 1.17–1.18 (d, J = 4.0 Hz, 3 H, CH(CH_3_)_2_). ^13^C NMR (CDCl_3_, ppm): δ 19.9 (CH_3_ of *p*-cymene), δ 24.7 (2CH_3_ of *p*-cymene), δ 31.8 (CH of *p*-cymene), δ 83.7–90.8 (aromatic carbons of *p*-cymene), δ 101.9 and δ 103.1 (quaternary carbons of *p*-cymene), δ 122.6, δ 128.4, δ 129.0, δ 130.0, δ 131.4, δ 132.0 (aromatic carbons), δ 175.5 (C = S), δ 162.1 (C = N), δ 147.9 (C-O), δ 41.2 (OCH_3_), δ 26.9 and δ 35.4 (NH-CH_2_CH_3_).

#### Synthesis of [Ru(*ƞ*^6^-*p*-cymene)(MSal-ptsc)Cl].Cl

Complex **4** was prepared by the procedure as described for (**1**) with 3-methoxysalicylaldehyde- 4(N)-phenylthiosemicarbazone [H_2_-Msal-ptsc] (0.049 g, 0.1632 mmol). The reddish orange suspension gradually turned brown color. The solvent was removed under reduced pressure. The complex was recrystallized from dissolved in CH_2_Cl_2_/hexane. The brown crystals were obtained. Yield: 89%. Mp. 173 °C. Anal. Calcd for C_25_H_31_Cl_2_N_3_O_3_RuS: C, 49.42; H, 4.81; N, 6.92; S, 5.28. Found: C, 49.39; H, 4.84; N, 6.90; S, 5.30%. FT-IR (cm^−1^) in KBr: 3427 (ʋ_O-H_), 1574 (ʋ_C = N_), 771 (ʋ_C=S_); UV-Vis (CH_2_Cl_2_), λ_max_: 246 (98,982) and 283 (109,327) nm (dm^3^ mol^−1^cm^−1^) (intra-ligand transition); ^1^H NMR (CDCl_3_, ppm): 10.68 (s, 1 H, –OH), 8.15 (s, 1 H, –CH = N), 6.98 (s, terminal –NH), 3.43 (s, 3 H, –OCH_3_), 9.18 (s, 1 H, –NH-C = S), 7.1–7.7 (m, aromatic), 6.29–6.30 (d, J = 4.0 Hz, 1 H, Hcymene), 5.99–6.01 (d, J = 6.0 Hz, 1 H, Hcymene), 5.69–5.71 (d, J = 8.0 Hz, 1 H, Hcymene), 5.43–5.44 (d, J = 4 Hz, 1 H, Hcymene), 3.91–3.99 (pent, J = 7.0 Hz, 1 H, CH(Me)_2_), 1.55 (s, 3 H, CH_3_-cymene), 0.79–0.81 (d, J = 8.0 Hz, 3 H, CH(CH_3_)_2_). ^13^C NMR (CDCl_3_, ppm): δ 14.9 (CH_3_ of *p*-cymene), δ 24.1 (2CH_3_ of *p*-cymene), δ 32.6 (CH of *p*-cymene), δ 83.3–86.9 (aromatic carbons of *p*-cymene), δ 102.2 and δ 106.1 (quaternary carbons of *p*-cymene), δ 127.3, δ 128.2, δ 131.9, δ 133.3, δ 136.3, δ 137.1 (aromatic carbons), δ 177.9 (C = S), δ 163.0 (C = N), δ 150.4 (C-O).

### DPPH radical scavenging activity

The antioxidative activities of ligands, starting precursor and complexes were determined by *in vitro* 2,2-diphenyl-1-picrylhydrazyl (DPPH) radical quenching experiment using UV-Vis spectrophotometer (UV-1800, Shimadzu, Japan) as described in our previous study^46^. The scavenging potential (IC_50_ - inhibition concentration) was calculated using the following formula:

$${\rm{DPPH}}\,{\rm{scavenging}}\,{\rm{effect}}\,( \% )=({\rm{Control}}\,{{\rm{OD}}}_{517nm}-{\rm{test}}\,{{\rm{OD}}}_{517nm}/{\rm{Control}}\,{{\rm{OD}}}_{517nm})\times 100$$

### *C. elegans*: genotype and maintenance

The following *C. elegans* strains were used: Bristol N2 (wild), EG1285 (*oxIs1*2 (*unc-47p::GFP* + *lin-15*)), TK22 (*mev-1* (*kn1*)), GR1307 (*daf-16* (*mgDf50*)), VC8 (*jnk-1*(*gk7*)), TJ356 (*zls356* (*daf-16::GFP*)), CF1553, (*muIs84* (*sod-3::*GFP)), CL2070 (*dvIs70* (*hsp-16.2p::GFP*), CL2166 (*dvIs19* (*gst-4p*::*GFP*)), GA800 (*wuIs151* (*ctl-1,2,3::GFP*)), HA759 (*rtIs11* (*osm-10p*::GFP + osm-10p::HtnQ150 + dpy-20)), AM141 (*rmIs133* [*unc-54p*::Q40::YFP)). All the strains were maintained and assayed on nematode growth media (NGM) agar plates following standard procedures^92^. Isogenic animal populations (age-sorting) were obtained by treating the gravid adult worms with 5 M NaOH and 5% NaOCl^93^.

### Toxicity and intracellular ROS measurement

To check the toxicity, wild type *C. elegans* were exposed to different pharmacological doses of complexes 1–4 (0, 2, 6, 10, 14 and 18 μM) at 20 °C, as previously demonstrated by us^46^. In order to quantify the intracellular ROS generation, about 25~30 control and complexes 1–4 treated *C. elegans* were incubated in cell permeable, ROS specific fluorogenic probe 2′ 7′-dichlorodihydrofluorescein diacetate (H_2_DCF-DA, Sigma) for 30 mins in the dark. After incubation, the worms were immobilized with 25 mM sodium azide (NaN_3_) and placed on microscopic slide coated with 3% agarose gel. Imaging of randomly selected animals were taken under the fluorescence microscope (BX-41, Olympus, Japan) equipped with digital camera (E330, Olympus, Japan) and the green fluorescent intensity was quantified using Image J software (NIH, Bethesda, MD) by determining the mean pixel intensity^77^.

### Assessment of oxidative stress resistance

To assess the effect of complexes **1–4** on oxidative stress resistance in wild-type *C. elegans*, an intracellular redox cycler 5-hydroxy-1,4-naphthoquinone (Juglone, Sigma) was used. The age-sorted L1 stage populations of N2 worms (25~30 individuals/replicate) were exposed to complexes **1–4** and 0.1% DMSO (solvent control) at 20 °C. At late L4 stage (on 6^th^ day of adulthood), they were then transferred to fresh NGM plates containing 240 μM juglone. The viability of worms was scored after 5 h of the continuous exposure. Three independent trails were performed with appropriate replicates.

### *C. elegans* longevity analysis

For longevity analysis, age synchronized L1 stage worms (25~30 individuals/treatment) were raised on the NGM plates with and without complexes **1–4**. A total of 50 μM 5-fluoro-2′-deoxyuridine (FUdR) was added to each plate to prevent the progeny development. During the course of experiment, worms being tested were periodically transferred to new treatment plates to prevent contaminations and to avoid starvation. The live/dead worms were scored at regular interval of every 2–3 days until the end of life. N2, *mev-1*, *daf-16* and *mek-1* mutant worms were employed in the lifespan evaluation.

### Reporter gene expression

Age-synchronized L3 worms of transgenic strains stably expressing *daf-16*::GFP (TJ356), *sod-3*::GFP (CF1553), *hsp-16.2*::GFP (CL2070), *gst-4*::GFP (CL2166) and *ctl-1,2,3::GFP* (GA800) were treated with complexes **1–4**(10 μM) for 72 h at 20 °C. Epigallocatechin gallate (EGCG), a green tea polyphenol served as a positive control for the strains TJ356, CF1553, CL2166 and GA800. Whereas, a short thermal stress at 37 °C for 2 h/EGCG was applied as positive control for strain CL2070. A total of 20~30 individuals/treatment were imaged in three independent experiments and analysed for GFP intensity as said above in clause Toxicity and intracellular ROS measurement.

### Neuronal survival assay

The transgenic strain HA759 expressing PolyQ tract in ASH neurons was used to detect the neuroprotective effects of Ru(*ƞ6-p*-cymene) complexes. Synchronized L1 stage worms of HA759 strain was grown on the NGM plates in the presence/absence of complexes **1–4** at 20 °C for 72 h. After that, control and treated *C. elegans* were placed on a microscopic slide and imaged under fluorescence microscope. About 20~30 randomly selected individuals/treatment were scored for GFP positive/negative in ASH neurons. For chemosensory behaviour assay, the experimental method was taken from Yang^94^.

### Assay for PolyQ aggregation

PolyQ aggregation assay was performed using AM141 *C. elegans* expressing polyQ40::YFP fusion protein in muscle cells, as described earlier^84^. Briefly, age sorted L1 worms were continuously exposed to complex **1–4** at 20 °C for indicated period of time. Thereafter, GFP images were taken using fluorescence microscope and the polyQ40::YFP aggregates in muscle cells of AM141 worms were quantified.

### Statistical analysis

Statistical analysis was performed using IBM SPSS Statistics for Windows, Version 21.0 (IBM Corporation, Armonk, NY, USA) and Excel, Microsoft office 2010 (MicrosoftCorporation, Redmond, WA, USA). For the lifespan assessment, survival of *C. elegans* was estimated using Kaplan-Meier survival curves and analysed by the log-rank test using MedCalc software, Version 14. Means were compared with untreated/DMSO treated control group using one way analysis of variance (ANOVA) followed by Bonferroni test (posthoc), values of *P* < 0.05 were considered as statically significant.

## Electronic supplementary material

Electronic supplementary material
