# Supplementary material for: Organoruthenium(II) Complexes Ameliorates Oxidative Stress and Impedes the Age Associated Deterioration in Caenorhabditis elegans through JNK-1/DAF-16 Signalling
Source: Sci Rep. 2018 May 16;8:7688. doi: 10.1038/s41598-018-25984-7 (PMC5955923; doi:10.1038/s41598-018-25984-7)
Supplement: Supplementary file 1 — Electronic supplementary material [file 41598_2018_25984_MOESM1_ESM.pdf]

Electronic supplementary material

Organoruthenium(II) Complexes Ameliorates Oxidative Stress and Impedes the Age Associated Deterioration in *Caenorhabditis elegans* through JNK-1/DAF-16 Signalling

G. Devagi [a], A. Mohankumar [b], G. Shanmugam [b], S. Nivitha [c], F. Dallemer [d], P. Kalaivani [e*], P. Sundararaj [b], R. Prabhakaran [a*]

[a] *Department of Chemistry, Bharathiar University, Coimbatore 641 046, India*
[b] *Department of Zoology, Bharathiar University, Coimbatore 641 046, India*
[c] *College of Science, Northeastern University, Boston, Massachusetts 02115, USA*
[d] *Laboratoire MADIREL CNRS UMR7246 , Université of Aix-Marseille , Centre de Saint-Jérôme, bât. MADIREL, 13397 Marseille Cedex 20, France*
[e] *Department of Chemistry, Nirmala College for Women, Bharathiar University, Coimbatore 641018*

***Correspondence to**

Dr. R. Prabhakaran
Assistant Professor
Department of Chemistry
Bharathair University
Coimbatore, Tamilnadu – 641046, India.
E-mail: rpnchemist@gmail.com
Mobile: +91-9442640334

Methods

Synthesis of ligands

Synthesis of 3-methoxysalicylaldehyde thiosemicarbazone[H_2-Msal-tsc] (H_2L^1)

Thiosemicarbazide (0.92 g, 10 mmol) was dissolved in 40 mLof methanol with continuous stirring and it was gently heatedfor a period of 30 min. To this, methanolic solution (10 mL) of 3-methoxy salicylaldehyde (1.53 g, 10 mmol) was added andthe mixture was refluxed by stirring for 2 h. Upon cooling, awhite crystalline product begins to separate. This was collected byfiltration, washed well with cold methanol and dried in vacuum.The product dissolves in common organic solvents such asacetone, methanol, ethanol, dichloromethane, chloroform, DMFand DMSO. Yield: 38%. Anal.calcd for $C_9H_{11}N_3O_2S$: C 47.98;H 4.92; N 18.65; S 14.23. Found: C 47.76; H 5.00; N 18.67; S14.20%. FT-IR (cm^{-1}) inKBr: 3458 (υ_{OH}), 1593 ($\upsilon_{C=N}$), 1272 ($\upsilon_{C–O}$), 818 ($\upsilon_{C=S}$); ^1H NMR (DMSO-d^6, ppm): 11.36 (s, 1H, OH), 9.13 (s, 1H, NHCS), 8.38 (s, 1H, CH=N), 7.84 and 8.06 (2br s, 1H each,NH_2), 3.79 (s, 3H, OCH_3), 6.73–7.52 (m, 3H, aromatic).

A similar method as described above was followed for thepreparation of all other thiosemicarbazone ligands.

3-methoxysalicylaldehyde-4(N)-methylthiosemicarbazone[H_2-Msal-mtsc] (H_2L^2)

The ligand [H_2-Msal-mtsc] was prepared from 4(N)-methylthiosemicarbazide (1.05 g, 10 mmol) and 3-methoxysalicylaldehyde (1.53 g, 10 mmol). Yield: 58%. Anal.Calcd for $C_{10}H_{13}N_3O_2S$: C,50.19; H, 5.47; N, 17.56; S, 13.40.Found: C, 50.15; H, 5.40; N, 17.49; S, 13.31%. FT-IR (cm^{-1}) inKBr: 3338 (υ_{OH}), 1554 ($\upsilon_{C=N}$), 1276 ($\upsilon_{C–O}$), 808 ($\upsilon_{C=S}$); ^1H NMR(DMSO-d^6, ppm): 11.40 (s, 1H, OH), 9.13 (s, 1H, NHCS), 8.37(s, 1H, $NHCH_3$), 8.36 (s, 1H, CH=N), 3.80 (s, 3H, OCH_3),6.75–7.54 (m, 3H, aromatic), 2.99 (d, 3H, CH_3).

3-methoxysalicylaldehyde-4(N)-ethylthiosemicarbazone[H_2-Msal-etsc] (H_2L^3)

The ligand [H_2-Msal-etsc] was prepared from 4(N)-ethylthiosemicarbazide (1.19 g, 10 mmol) and 3-methoxysalicylaldehyde (1.53 g, 10 mmol). Yield: 64%. Anal.calcd for $C_{11}H_{15}N_3O_2S$: C 55.21; H 6.31; N 16.58; S 12.65. Found:C 55.15; H 6.27; N 16.50; S 12.59%. FT-IR (cm^{-1}) in KBr: 3310(υ_{OH}), 1536 ($\upsilon_{C=N}$), 1276 ($\upsilon_{C–O}$), 818 ($\upsilon_{C=S}$); ^1H NMR (DMSO-d^6,ppm): 11.34 (s, 1H, OH), 9.14 (s, 1H, NHCS), 8.42 (s, 1H,NHC_2H_5), 8.40 (s, 1H, CH=N), 3.80 (s, 3H, OCH_3), 6.75–7.53(m, 3H, aromatic), 3.55–3.58 (m, 2H, CH_2), 1.13 (t, 3H, CH_3).

3-methoxysalicylaldehyde- 4(N)-phenylthiosemicarbazone[H_2-Msal-ptsc] (H_2L^4)

The ligand [H_2-Msal-ptsc] was prepared from 4(N)-phenylthiosemicarbazide (1.67 g, 10 mmol) and 3-methoxysalicylaldehyde (1.53 g, 10 mmol). Yield: 54%. Anal.calcd for $C_{15}H_{15}N_3O_2S$: C 59.81; H 5.12; N 13.95; S 10.64. Found:C 59.65; H 4.99; N 13.78; S 10.43%. FT-IR (cm^{-1}) in KBr: 3339(υ_{OH}), 1589 ($\upsilon_{C=N}$), 1273 (υ_{C-O}), 782 ($\upsilon_{C=S}$); ^{1}H NMR (DMSO-d^6,ppm): 11.76 (s, 1H, OH), 10.00 (s, 1H, NHCS), 9.20 (s, 1H,NHPh), 8.50 (s, 1H, CH=N), 3.81 (s, 3H, OCH_3), 6.76–7.68 (m,8H, aromatic).

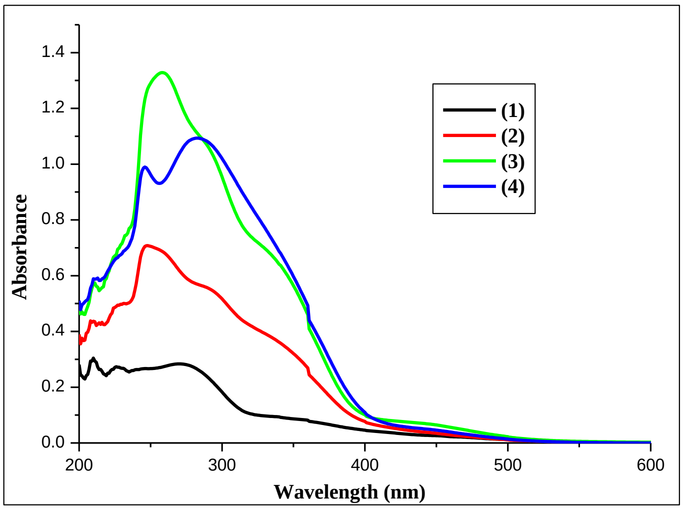

Figure S1. Electronic absorption spectrum of complexes (**1-4**)

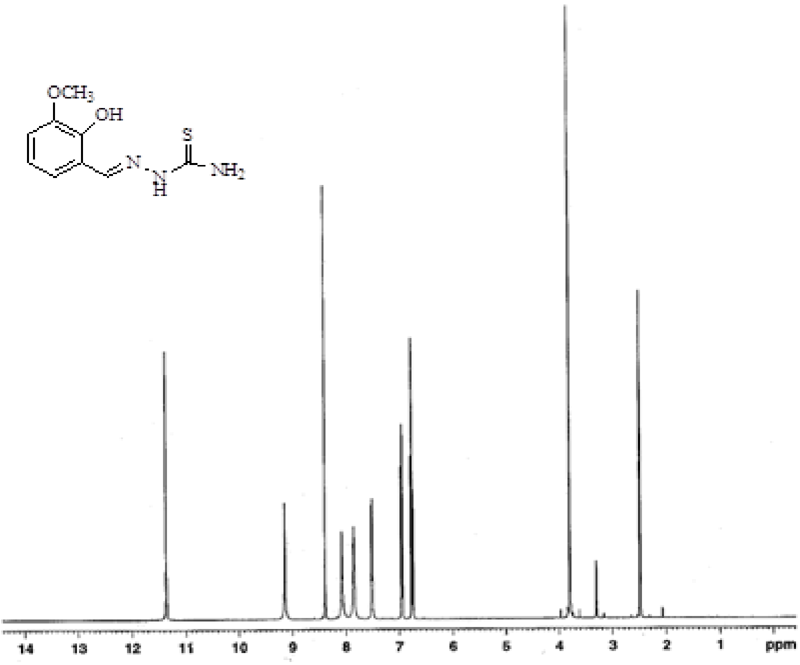

Figure S2.^{1}H NMR spectrum of [H_2-Msal-tsc] (**H_2L^1**)

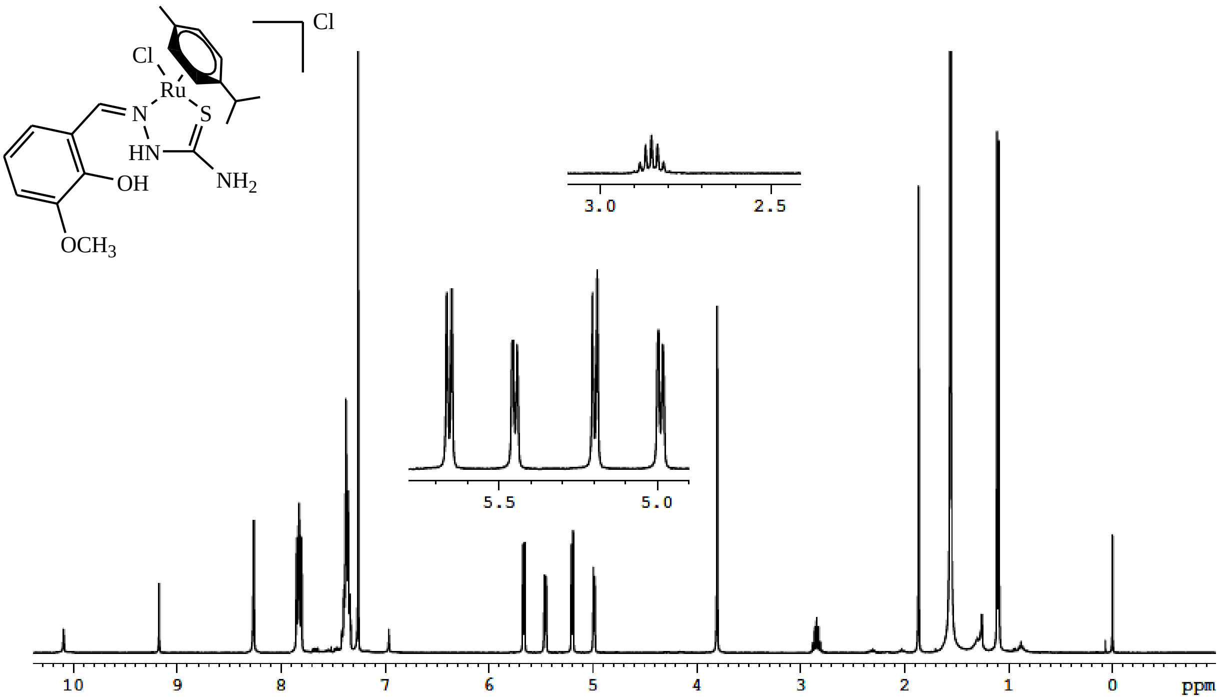

Figure S3.^{1}H NMR spectrum of [Ru(η^6-*p*-cymene)(MSal-tsc)Cl].Cl (**IV. 1**)

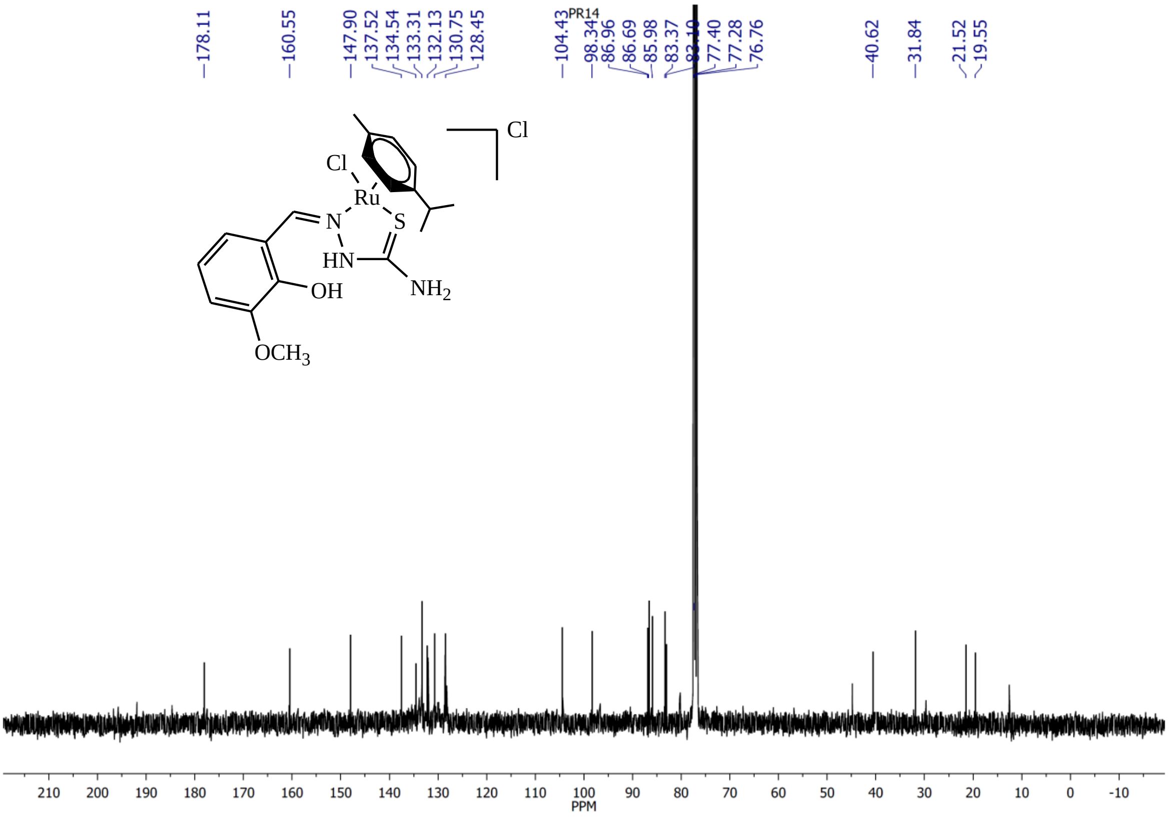

Figure S4.^{13}C NMR spectrum of [Ru(η^6-*p*-cymene)(MSal-tsc)Cl].Cl **(IV. 1)**

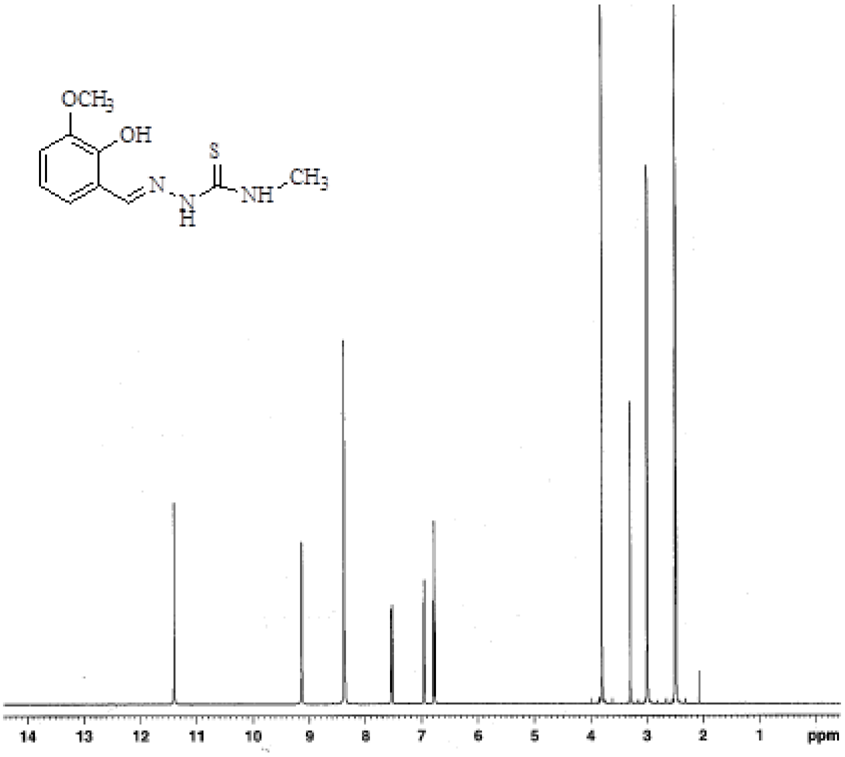

Figure S5. ^{1}H NMR spectrum of [H_2-Msal-mtsc] **(H_2L^2)**

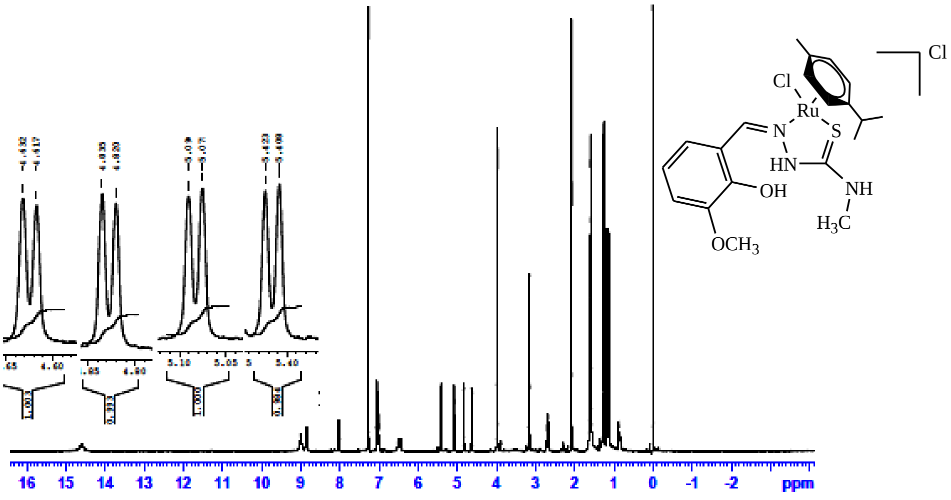

Figure S6. ^{1}H NMR spectrum of [Ru(η^6-*p*-cymene)(MSal-mtsc)Cl].Cl **(IV. 2)**

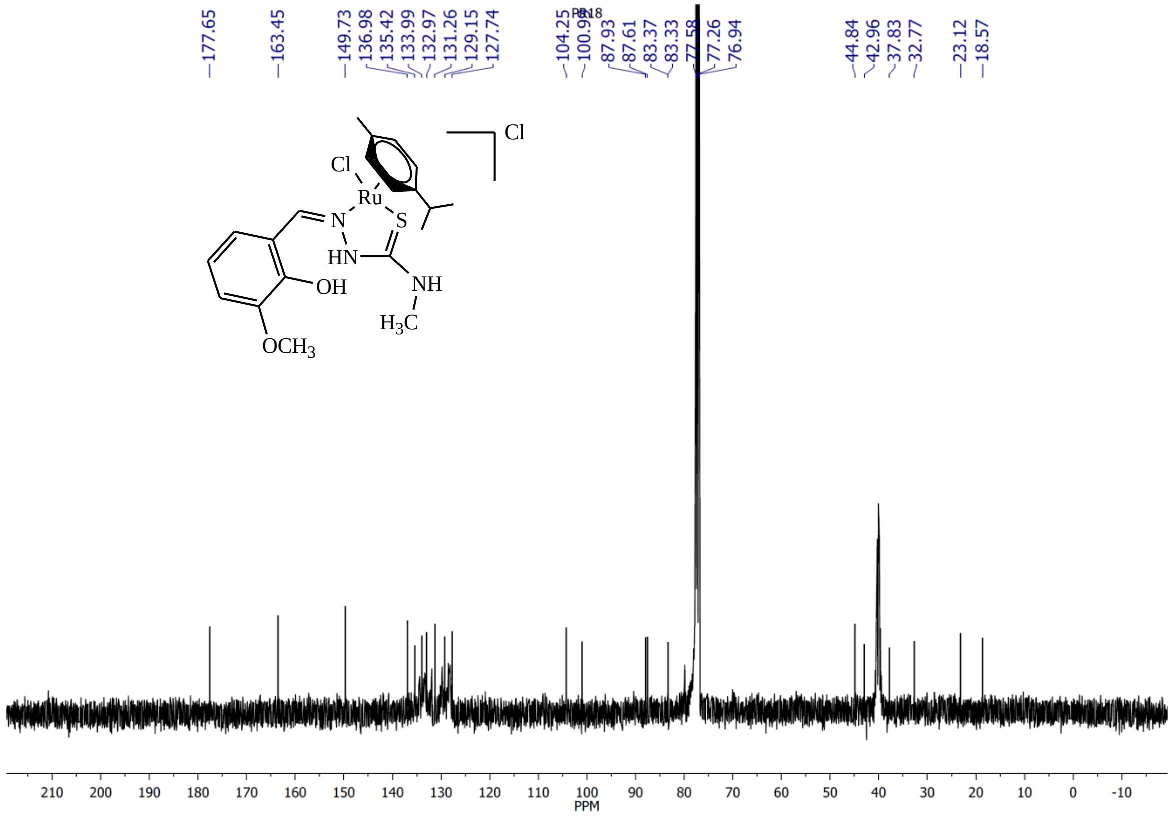

Figure S7.^{13}C NMR spectrum of [Ru(η^6-*p*-cymene)(MSal-mtsc)Cl].Cl (**IV. 2**)

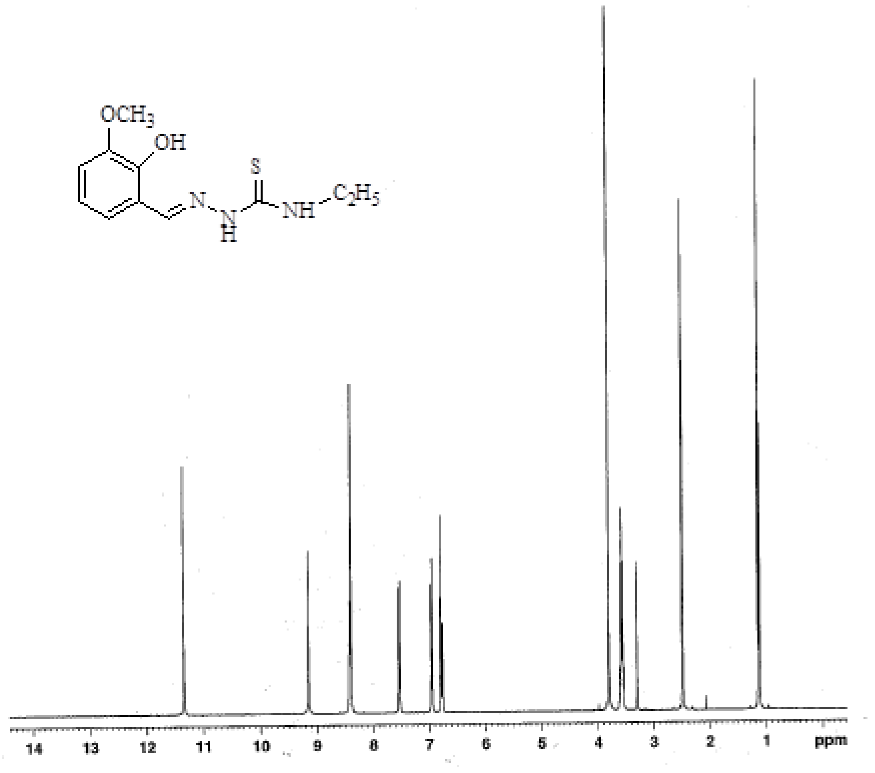

Figure S8. ^{1}H NMR spectrum of [H_2-Msal-etsc] **(H_2L^3)**

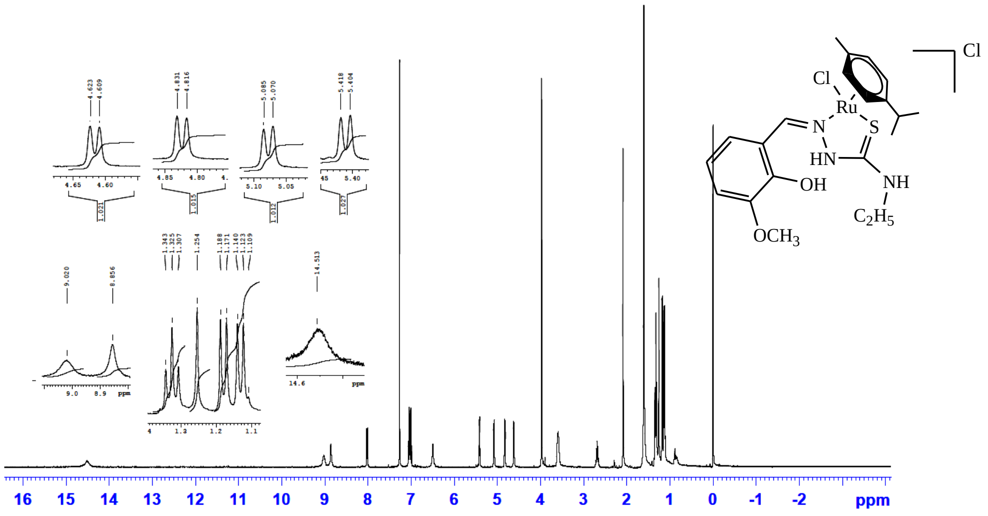

Figure S9.^{1}H NMR spectrum of [Ru(η^6-*p*-cymene)(MSal-etsc)Cl].Cl **(IV. 3)**

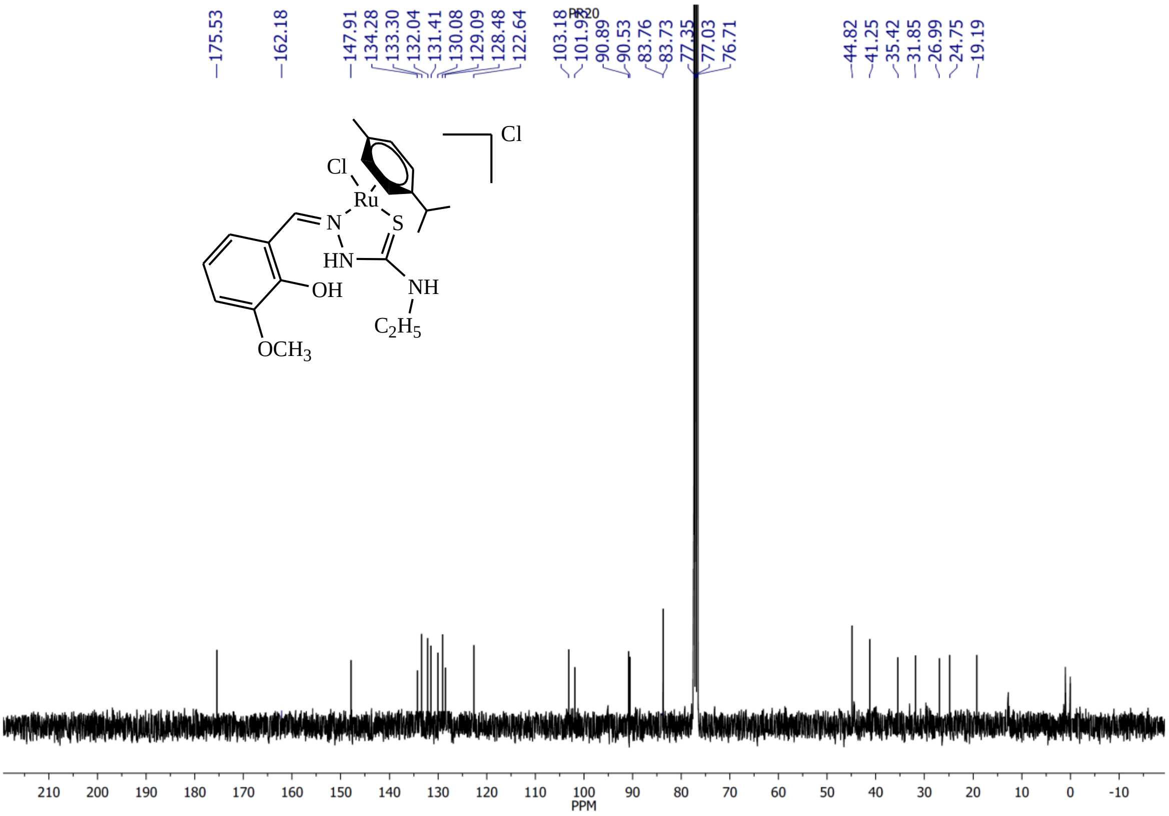

Figure S10.^{13}C NMR spectrum of [Ru(η^6-*p*-cymene)(MSal-etsc)Cl].Cl (**IV. 3**)

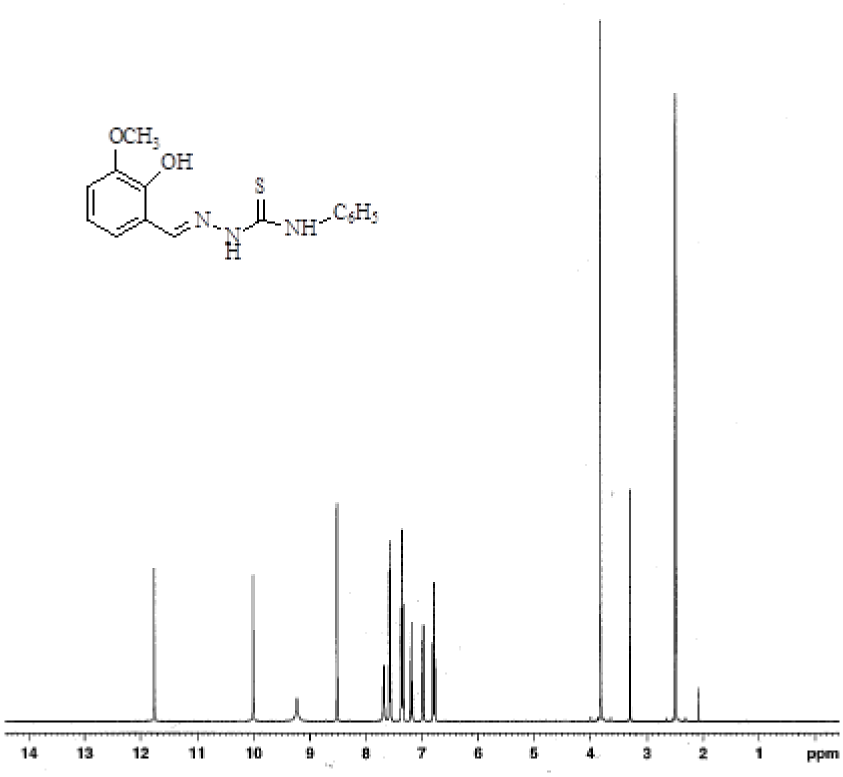

Figure S11. ^{1}H NMR spectrum of [H_2-Msal-ptsc] **(H_2L^4)**

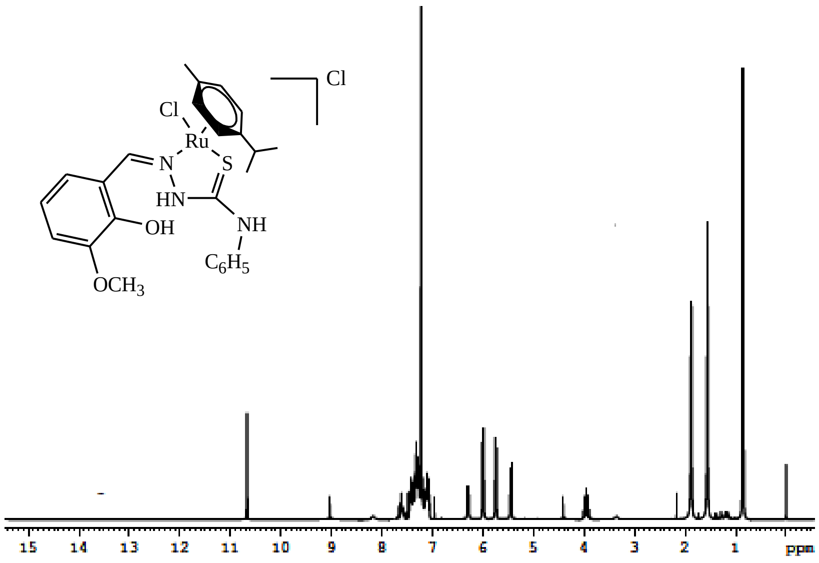

Figure S12.^{1}H NMR spectrum of [Ru(η^6-*p*-cymene)(MSal-ptsc)Cl].Cl **(IV. 4)**

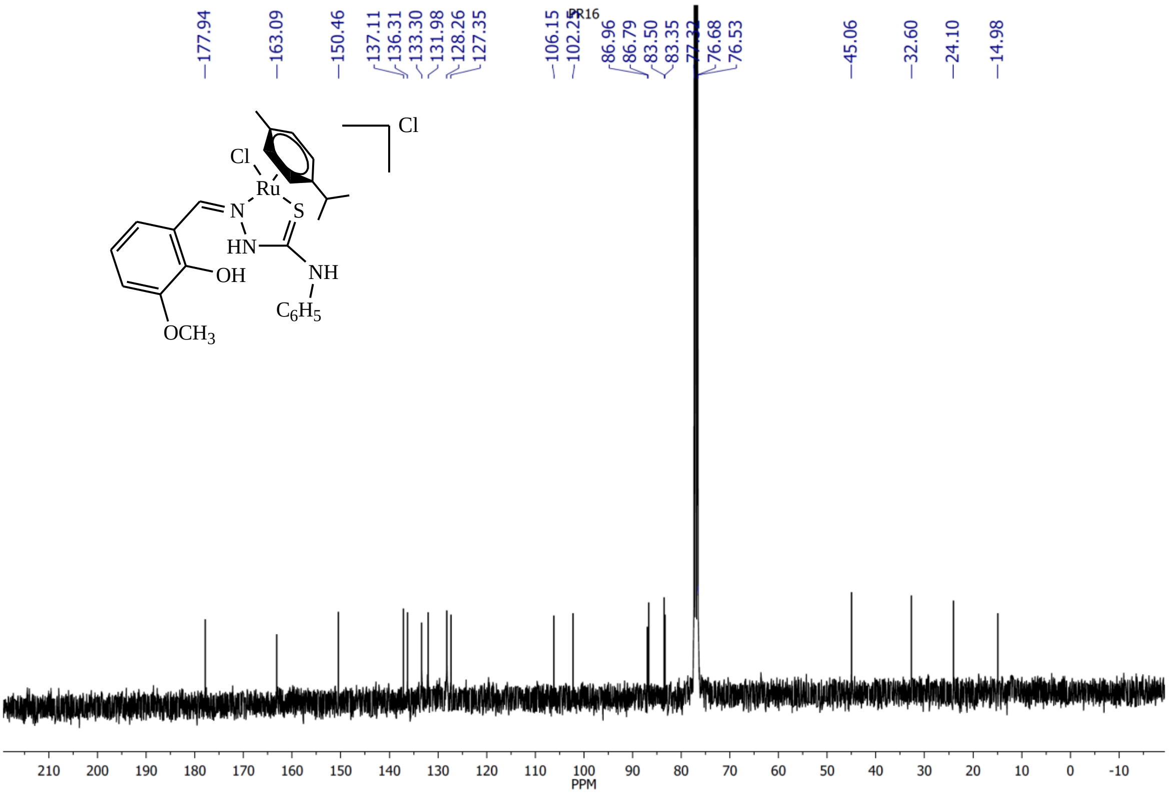

Figure S13.^{13}C NMR spectrum of [Ru(η^6-*p*-cymene)(MSal-ptsc)Cl].Cl **(IV. 4)**

^{1}H-NMR and ^{13}C-spectroscopy

In the ^{1}H NMR spectra showed a loss of the two fold symmetry of the *p*-cymene ligand, similar to other reported *p*-cymene complexes[1,2].The four aromatic hydrogens of the *p*-cymene ligand appear as four sets of doublets in the ^{1}H-NMR spectra in the range of δ 4.98-5.71 ppm. The most distinctive feature of the *p*-cymene ligand is that the pentet between δ 2.81 and δ 4.0 ppm, which is due to the single CH proton of the isopropyl group. The methyl group protons of the same group give rise to a doublet between δ 0.8 and δ 1.18 ppm. A singlet between δ 1.25 and δ 1.86 ppm can be assigned to the methyl group that is attached directly to the aromatic ring. In the spectra of H_2L^1–H_2L^4, a singlet appeared in the range δ 9.13–10.00 ppm has been assigned to N(2)HCS group[3] were appeared in the complex at δ 8.99-9.18 ppm indicating the coordination of sulphur atom to the ruthenium in the thione form[4].A sharp singlet appeared at δ 11.34–11.76 ppm corresponding to the phenolic –OH group in the free ligands was appeared in the complex at δ 10.1-14.58 ppm indicating the non participation of phenolic oxygen in coordination[5]. The aromatic protons corresponding to the presence of coordinated ligands were appeared as multiplet at δ 7.00-7.77 ppm, and a singlet

corresponding to the–OCH_3 group also found at δ 3.61– 3.97 ppm[6]. A singlet observed at δ 8.36–8.50 ppm in the ligand was appeared at δ 8.00-8.85 ppm in the complexes has been assigned to azomethine proton[6]. Singlet at δ 8.37– 9.20 ppm in the ligand was assigned to the terminal –NH protons of the ligands which appeared in the complex as a singlet at δ 6.9 ppm for **1**and **4**, as a multiplet at δ 8.0 ppm for complex **2** and as a doublet at δ 8.0 ppm for **3** was observed[6]. In the spectra of $\mathbf{H_2L^2}$ doublet appeared at 2.99 ppm was appeared as a singlet at δ 2.08 ppm in the complex **2** corresponding to the presence of terminal CH_3 protons and a triplet observed at δ 1.13 ppm and δ 1.34 ppm corresponding to a terminal CH_3 group of $\mathbf{H_2L^3}$ and complex **3**. In addition, a multiplet appeared at δ 3.55 ppm in **H2L3** was appeared as doublet at δ 3.58 ppm in the complex **3** has been assigned to the terminal methylene protons of ethyl group[6]. The ^{13}C {1H} NMR spectra of the complexes (**1-4**) contain resonances for the aromatic carbons around δ 122.6-137.5 ppm[7]. The signals observed around δ 160.5-163.4 ppm assigned to the azomethine (HC=N) carbon of the ligands. [8] The resonance due to (C-O) and (C=S) were observed around δ 147.9-150.4 ppm and δ 175.5-178.1 ppm, respectively. The signal observed around δ 40.6-42.9 ppm due to the presence of methoxy carbon of the ligands. In all the complexes (**1-4**), the signals observed around δ 14.9-19.9, δ 21.5-24.7, δ 31.8-32.7, δ 83.1-90.8, δ 98.3-102.2 and δ 103.1-106.1 ppm confirms the presence of *p*-cymene carbon atom[7,-9]. In complex **2**, a signal observed at δ 37.83 ppm has been assigned to terminal NH-CH_3 carbon[10].In complex **3**, signals observed at δ 26.9 ppm and δ 35.4 ppm were corresponding to the presence of methylene and methyl carbons of ethyl group[10].

References

1. Hamaker, C.G. *et al*. Synthesis, structure, and characterization of some ruthenium arene complexes of N-(arylmethylene)-2-(methylthio)anilines and 2-(methylthio)aniline. *Inorg. Chim. Acta* **359**, 846-852 (2006).
2. Moldes, I. *et al*. Ruthenium(II) complexes containing both arene and functionalized phosphines. Synthesis and catalytic activity for the hydrogenation of styrene and phenylacetylene. *J. Organomet. Chem.* **566**, 165-174 (1998).
3. Lobana, T.S.*et al*. Symmetrisation, isomerism and structural studies on novel phenylmercury(II) thiosemicarbazonates: correlation of the energy barrier to rotation of the amino group with the bonding parameters of the thioamide group. *Dalt. Trans.*, 4289-4299(1997).
4. Muhammad, H. *et al*. Ruthenium(II) (η^6-arene) Complexes of thiourea derivatives: Synthesis, characterization and urease inhibition, *Molecules* **19**, 8080-8092(2014).
5. Basuli, F. *et al*. Unusual coordination mode of thiosemicarbazone ligand. Synthesis, structure and redox properties of some ruthenium and osmium complexes. *Inorg. Chem.* **37**, 6113-6116(1998).

6. Kalaivani, P. *et al*. Influence of terminal substitution on structural, DNA, Protein binding, anticancer and antibacterial activities of palladium(II) complexes containing 3-methoxy salicylaldehyde-4(N) substituted thiosemicarbazones. *Dalt. Trans*. **41**, 2486-2499(2012).
7. Sheeba, M. M. *et al*. Chiral (η^6-*p*-Cymene) ruthenium(II) complexes containing monodentate acylthiourea ligands for efficient asymmetric transfer hydrogenation of ketones. *Organometallics* **33**, 540-550 (2014).
8. Kalaivani, P. *et al*. Versatile Coordination Behavior of Salicylaldehyde thiosemicarbazone in Ruthenium(II) Carbonyl Complexes: Synthesis, Spectral, X-ray, Electrochemistry, DNA Binding, Cytotoxicity, and Cellular Uptake Studies. *Organometallics* **31**, 8323-8332(2012).
9. Grabulosa, A. *et al*. Neutral *p*-cymene ruthenium complexes with P-stereogenicmonophosphines. New catalytic precursors in enantioselective transfer hydrogenation and cyclopropanation. *J. Organomet. Chem*. **696**, 4221-4228(2012).
10. Devagi, G. *et al*. *Caenorhabditis elegans* as a model for exploring the efficacy of synthesized organoruthenium complexes for aging and Alzheimer's disease a neurodegenerative disorder: A systematic approach. *J. Organomet. Chem*. **838**, 12–23(2017)

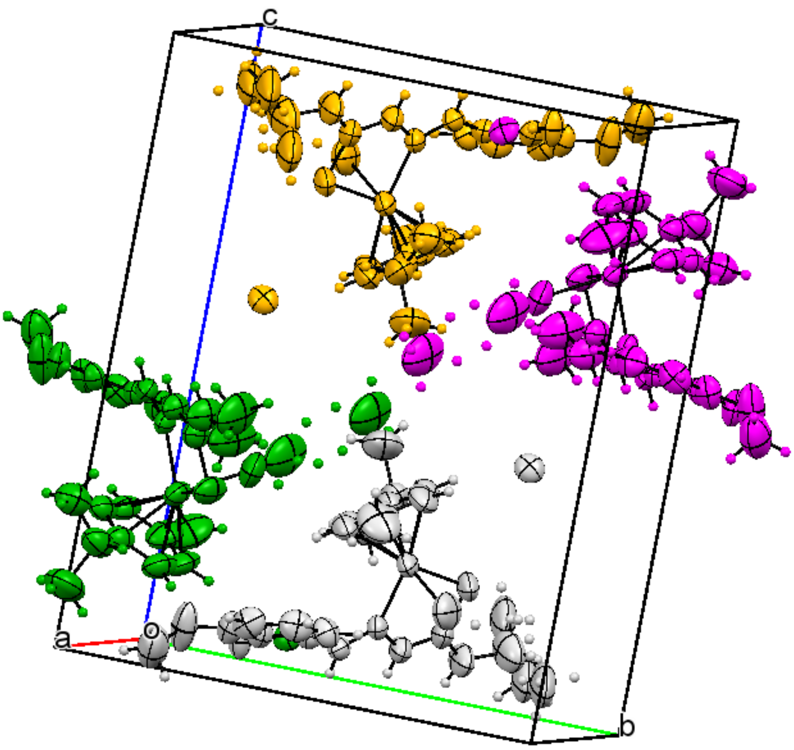

Figure S14. Molecular packing diagram of [Ru(η^6-*p*-cymene)(MSal-etsc)Cl].Cl **(IV. 3)**

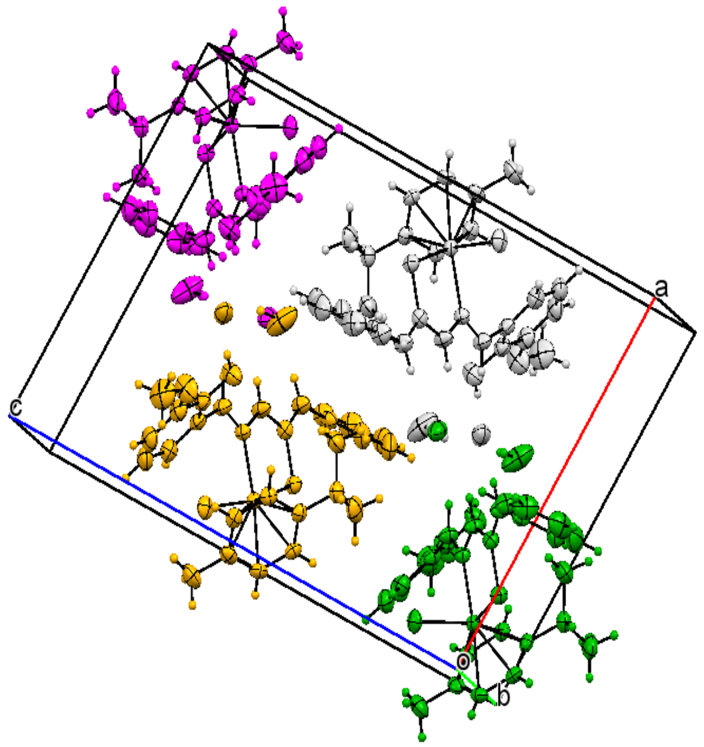

Figure S15. Molecular packing diagram of [Ru(η^6-*p*-cymene)(MSal-ptsc)Cl].Cl **(IV. 4)**

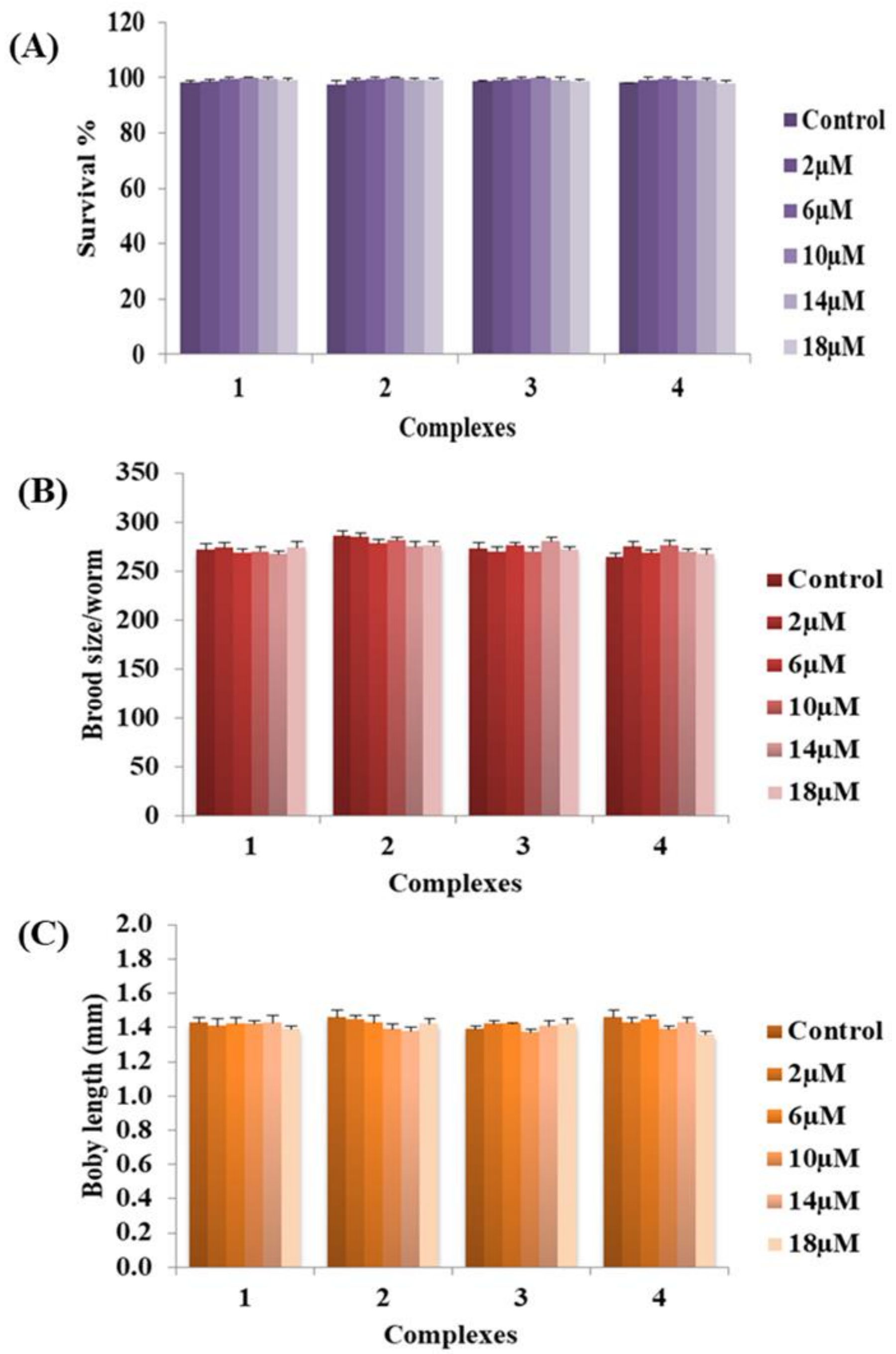

Figure S16. Effect of complexes **1-4** on *C. elegans* survival, reproduction and development. Different pharmacological doses of Ru(η6-*p*-cymene) complexes did not obviously alters the (**A**) survival rate (top), (**B**) progeny production (middle) and (**C**) development (bottom) in *C. elegans*. Bar graphs are expressed as mean±SEM of three independent experiments. *P* values were calculated by Bonferroni post hoc test. $^{**}P<0.001$.

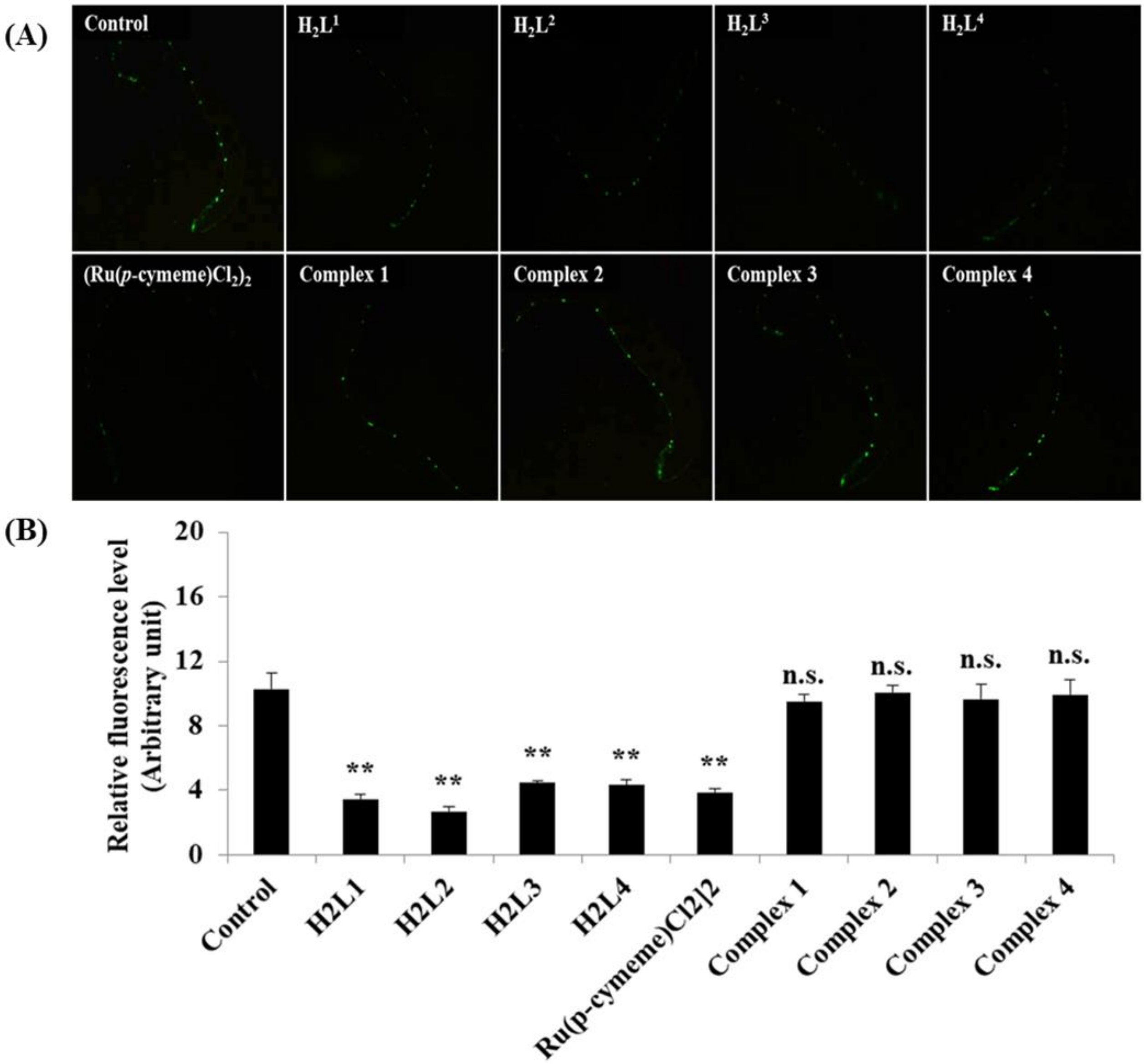

Figure S17. Effect of ligands, starting precursor and complexes on the development/morphology of D-type GABAergic motor neurons in EG1285 *C. elegans*. (**A**) Fluorescent intensity of cell bodies at ventral cord of D-type GABAergic motor neurons treated with ligands, starting precursor and complexes was measured using image J software by determining the mean pixel intensity. (**B**) Bar graphs are expressed as mean±SEM of three independent experiments (n=20/experiment). *P* values were calculated by Bonferroni post hoc test, $^{n.s.}$not significant and $^{**}P<0.001$.

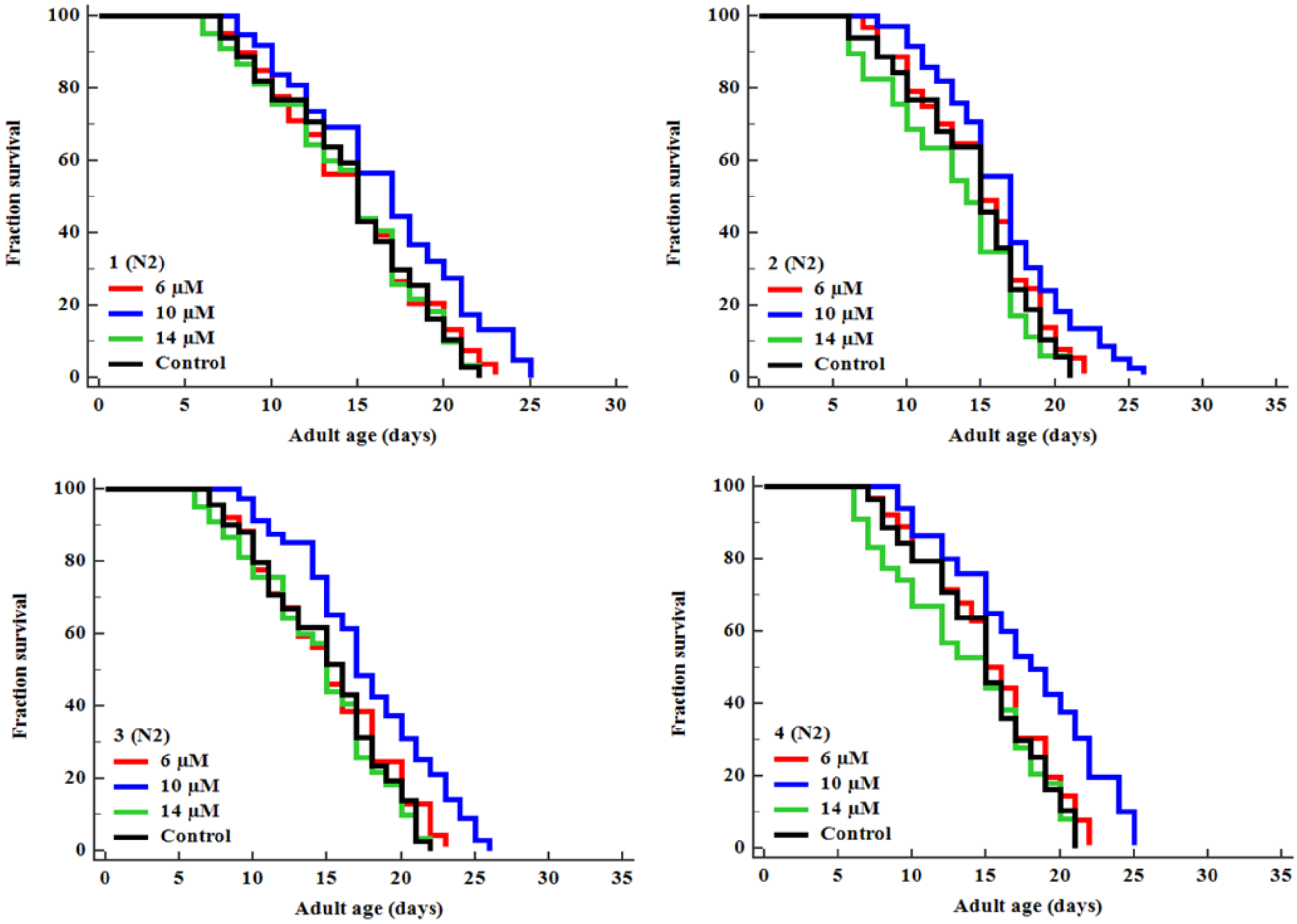

Figure S18. New Ru(*η6-p*-cymene) complexes at 10 µM concentration significantly ($p<0.0001$) extended the lifespan of N2 *C. elegans* under standard laboratory conditions. Survival differences of *C. elegans* was estimated using Kaplan-Meier survival curves and analyzed by the log-rank test using MedCalc software.

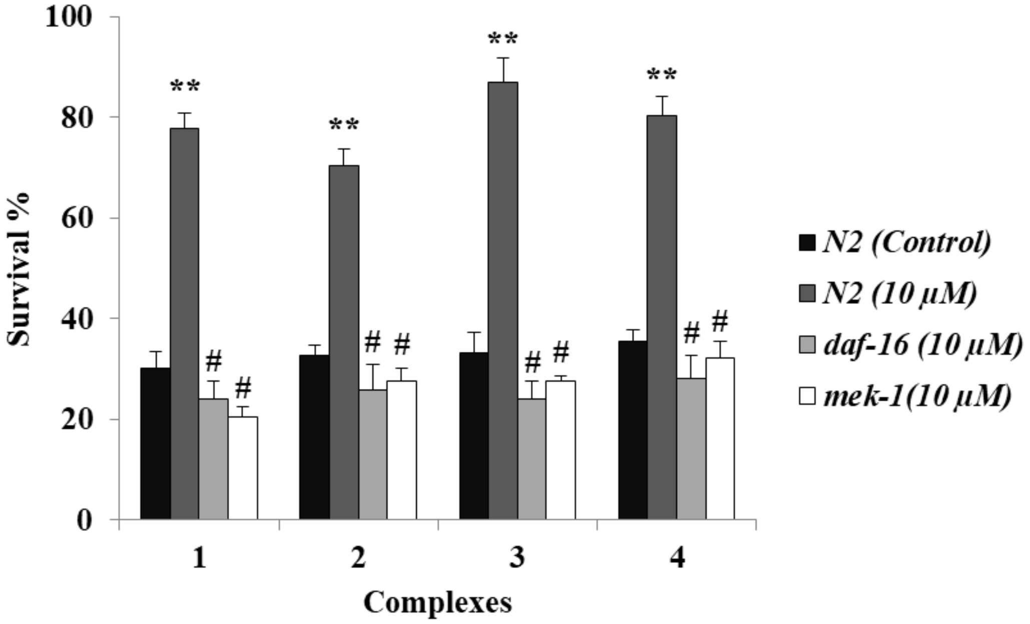

Figure S19. Protective effect of Ru(*η6-p*-cymene) complexes depends on *daf-16* and *jnk-1* in *C. elegans*. In contrast to the result obtained with wild-type *C. elegans*, all complexes failed to increase the survival rate of *daf-16* and *mek-1* mutant worms under juglone exposed condition. Bar graphs are expressed as mean±SEM of three independent experiments (n=20~25 individuals/replicate). *P* values were calculated by Bonferroni post hoc test, $^{**}P<0.001$ (in comparison with untreated wild-type) and $^{\#}P<0.001$ (in comparison with wild-type).

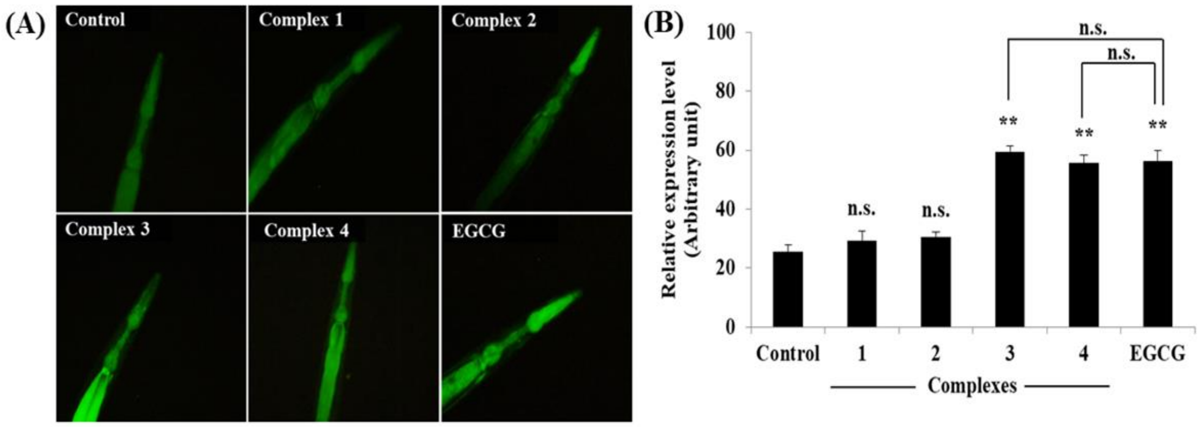

Figure S20. Ru(*η6-p*-cymene) complexes induce the expression of *hsp-16.2::GFP* transgene in *C. elegans*. (**A**) Images of worms treated with 0.1 % DMSO (control), complexes **1-4** or EGCG (positive control). (**B**) Quantification of GFP intensity in the worm's pharynx of control and treated groups. Data are represented as mean ± SEM of three independent runs (n=20~30 individuals/experiments), *P* values were calculated by Bonferroni post hoc test. $^{n.s.}$not significant, $^{*}P<0.05$ and $^{**}P<0.001$.

Table S1. Selected bond lengths (**Å**) and angels(°)for Complex **3** and **4.**

	**Complex IV. 3**	**Complex IV. 4**
Ru1-Cl1	2.3898(16)	2.4055(4)
Ru1-S1	2.3623(14)	2.3519(4)
Ru1-N1	2.113(3)	2.1200(14)
Ru1-C13	2.244(6)	-
Ru1-C14	2.171(5)	-
Ru1-C15	2.167(5)	-
Ru1-C16	2.201(5)	2.2031(17)
Ru1-C17	2.181(5)	2.1698(17)
Ru1-C18	2.225(5)	2.1829(17)
Ru1-C19	-	2.2649(17)
Ru1-C20	-	2.2496(17)
Ru1-C21	-	2.1696(17)

	**Complex 3**		**Complex 4**
S1-Ru1-Cl1	86.23(6)	S1-Ru1-Cl1	88.376(16)
N1-Ru1-Cl1	86.16(10)	N1-Ru1-Cl1	83.58(4)
N1-Ru1-S1	82.27(9)	N1-Ru1-S1	82.06(4)
N1-Ru1-C13	128.86(19)	N1-Ru1-C16	111.21(6)
N1-Ru1-C14	166.25(19)	N1-Ru1-C17	146.54(6)
N1-Ru1-C15	148.8(2)	N1-Ru1-C18	169.53(7)
N1-Ru1-C16	112.62(16)	N1-Ru1-C19	132.36(6)
N1-Ru1-C17	92.73(15)	N1-Ru1-C20	103.73(6)
N1-Ru1-C18	100.74(15)	N1-Ru1-C21	93.59(6)
C13-Ru1-Cl1	91.40(19)	C16-Ru1-Cl1	165.20(5)
C13-Ru1-S1	148.60(16)	C16-Ru1-S1	93.11(5)
C14-Ru1-Cl1	94.7(2)	C16-Ru1-C19	80.32(7)
C14-Ru1-S1	111.48(18)	C16-Ru1-C20	67.55(6)
C14-Ru1-C13	37.5(2)	C17-Ru1-Cl1	127.54(5)
C14-Ru1-C16	68.2(2)	C17-Ru1-S1	86.47(5)
C14-Ru1-C17	80.0(2)	C17-Ru1-C16	38.06(7)
C14-Ru1-C18	66.5(2)	C17-Ru1-C18	37.70(7)
C15-Ru1-Cl1	122.83(19)	C17-Ru1-C19	67.24(7)
C15-Ru1-S1	87.92(17)	C17-Ru1-C20	78.62(7)
C15-Ru1-C13	67.2(2)	C18-Ru1-Cl1	96.80(5)
C15-Ru1-C14	37.3(2)	C18-Ru1-S1	108.41(5)
C15-Ru1-C16	37.9(2)	C18-Ru1-C16	68.76(7)
C15-Ru1-C17	67.60(19)	C18-Ru1-C19	37.31(7)
C15-Ru1-C18	78.3(2)	C18-Ru1-C20	66.27(7)
C16-Ru1-Cl1	160.74(14)	C19-Ru1-Cl1	90.05(5)
C16-Ru1-S1	91.92(14)	C19-Ru1-S1	145.10(5)
C16-Ru1-C13	80.2(2)	C20-Ru1-Cl1	110.47(5)
C16-Ru1-C18	66.7(2)	C20-Ru1-S1	160.65(5)
C17-Ru1-Cl1	151.53(15)	C20-Ru1-C19	36.04(7)
C17-Ru1-S1	121.87(15)	C21-Ru1-Cl1	146.14(5)
C17-Ru1-C13	67.3(2)	C21-Ru1-S1	124.77(5)
C17-Ru1-C16	37.61(17)	C21-Ru1-C16	37.75(7)
C17-Ru1-C18	36.77(18)	C21-Ru1-C17	67.70(7)
C18-Ru1-Cl1	115.58(18)	C21-Ru1-C18	80.23(7)
C18-Ru1-S1	158.06(17)	C21-Ru1-Cl9	67.10(7)
C18-Ru1-C13	36.8(2)	C21-Ru1-C20	37.48(6)
C10-S1-Ru1	98.71(17)	C1-S1-Ru1	99.63(6)

Table S2. Hydrogen Bonds for Complexes **3** and **4** (Å and deg)

		**Complex 3**[a]		
D—H···A	*d*(D—H)	*d*(H···A)	*d*(D···A)	∟(DHA)
O(1)—H(1)···Cl(2)	0.820	2.222	2.993	156.62
N(2)—H(2)···Cl(2)	0.860	2.286	3.092	156.18
N(3)—H(3)···Cl(2)	0.860	2.515	3.276	148.09
		**Complex 4**[b]		
O(1)—H(1)···Cl(2)	0.820	2.300	3.036	149.81
N(2)—H(2)···Cl(2)	0.861	2.403	3.224	159.50
N(3)—H(3)···O(3)	0.860	1.937	2.763(3)	160.53
O(3)—H(3A)···Cl(2)	0.993	2.221	3.203(2)	169.70
O(3)—H(3B)···Cl(2)	0.986	2.257	3.15(2)	151.25

[a]Symmetry operation: (x, y, z); (-½ +x,1.5-y, -½ + z), (1.5-x,- -½+y,-½ - z).

[b]Symmetry operation: (x, y, z);(x, y, z), (-x, ½+y, ½-z), (-x,-y,-z), (x,½-y,½+z)

Table S3. Longevity analysis: Effect of different pharmacological doses of new Ru(*η6-p*-cymene) complexes on mean lifespan of wild-type and mutant *C. elegans*.

Genotype	Complex	Treatment	Mean survival (days±SEM)	% Change	*P* Value
Wild	**1**	Control	14.583±0.398		
		6 µM	14.622±0.371	(+) 0.27	0.3082
		10 µM	16.489±0.402	(+) 13.07	0.0001
		14 µM	14.503±0.381	(-) 0.55	0.6008
	**2**	Control	14.514±0.388		
		6 µM	14.939±0.369	(+) 2.93	0.1944
		10 µM	16.349±0.333	(+) 12.64	0.0001
		14 µM	13.393±0.419	(-) 7.72	0.1475
	**3**	Control	14.999±0.380		
		6 µM	15.064±0.392	(+) 0.43	0.2779
		10 µM	17.547±0.336	(+) 16.99	0.0001
		14 µM	14.503±0.381	(-) 3.31	0.4604
	**4**	Control	14.901±0.388		
		6 µM	15.294±0.374	(+) 2.64	0.1487
		10 µM	17.371±0.370	(+) 16.58	0.0001
		14 µM	13.799±0.450	(-) 7.40	0.6375
mev-1	**1**	Control	10.294±0.324		
		6 µM	10.080±0.335	(-) 2.08	0.6057
		10 µM	11.591±0.268	(+)12.60	0.0001
		14 µM	10.813±0.316	(+) 5.04	0.1593
	**2**	Control	10.189±0.316		
		6 µM	10.121±0.342	(-) 0.67	0.5797
		10 µM	11.884±0.247	(+) 16.64	0.0001
		14 µM	11.001±0.376	(+) 7.97	0.0155
	**3**	Control	10.389±0.321		
		6 µM	10.295±0.339	(-) 0.90	0.4782
		10 µM	12.708±0.220	(+) 22.32	0.0001
		14 µM	10.733±0.338	(+) 3.31	0.2187
	**4**	Control	10.092±0.334		
		6 µM	10.813±0.321	(+) 7.14	0.0940
		10 µM	12.408±0.262	(+) 22.95	0.0001
		14 µM	11.223±0.350	(+) 11.21	0.0024
daf-16		Control	10.623±0.298		
	**1**	10 µM	10.454±0.278	(-) 1.59	0.3646
	**2**	10 µM	10.502±0.332	(-) 1.14	0.6589
	**3**	10 µM	10.239±0.287	(-) 3.61	0.1741
	**4**	10 µM	10.611±0.323	(-) 0.11	0.8355
mek-1		Control	12.635±0.365		
	**1**	10 µM	12.624±0.351	(-) 0.09	0.7770
	**2**	10 µM	11.856±0.297	(-) 6.17	0.0557
	**3**	10 µM	12.556±0.365	(-) 0.63	0.7912
	**4**	10 µM	12.698±0.295	(+) 0.50	0.9360

checkCIF () running

Checking for embedded fcf data in CIF ...
No extractable fcf data in found in CIF

checkCIF/PLATON (full publication check)

You have not supplied any structure factors. As a result the full set of tests cannot be run.

THIS REPORT IS FOR GUIDANCE ONLY. IF USED AS PART OF A REVIEW PROCEDURE FOR PUBLICATION, IT SHOULD NOT REPLACE THE EXPERTISE OF AN EXPERIENCED CRYSTALLOGRAPHIC REFEREE.You have not supplied any structure factors. As a result the full set of tests cannot be run.

```
No syntax errors found.
Please wait while processing ....
```

CIF c
Inter

Datablock: I

Bond precision:	C-C = 0.0078 A		Wavelength=0.71073
Cell:	a=9.9066(4)	b=14.3359(4)	c=17.9298(6)
	alpha=90	beta=92.228(4)	gamma=90
Temperature:	293 K		

	Calculated	Reported
Volume	2544.47(15)	2544.46(16)
Space group	P 21/n	P 1 21/n 1
Hall group	-P 2yn	-P 2yn
Moiety formula	C21 H28 Cl N3 O2 Ru S, Cl	C21 H28 Cl N3 O2 Ru S, Cl
Sum formula	C21 H28 Cl2 N3 O2 Ru S	C21 H28 Cl2 N3 O2 Ru S
Mr	558.49	558.49
Dx,g cm-3	1.458	1.458
Z	4	4

Mu (mm-1)	0.929	0.929
F000	1140.0	1140.0
F000'	1136.94	
h,k,lmax	13,19,24	13,19,23
Nref	6480	5893
Tmin,Tmax	0.837,0.911	0.695,1.000
Tmin'	0.815	

Correction method= # Reported T Limits: Tmin=0.695 Tmax=1.000 AbsCorr = MULTI-SCAN

Data completeness= 0.909 Theta(max)= 28.548

R(reflections)= 0.0615(3946) wR2(reflections)= 0.1162(5893)

S = 1.058 Npar= 282

The following ALERTS were generated. Each ALERT has the for
test-name_ALERT_alert-type_alert-level.
Click on the hyperlinks for more details of the test.

Alert level B

PLAT234_ALERT_4_B Large Hirshfeld Difference C11 -- C24 .. 0.27 Ang.

Alert level C

PLAT220_ALERT_2_C Large Non-Solvent C Ueq(max)/Ueq(min) Range 3.3 Ratio
PLAT222_ALERT_3_C Large Non-Solvent H Uiso(max)/Uiso(min) ... 4.2 Ratio
PLAT230_ALERT_2_C Hirshfeld Test Diff for O2 -- C8 .. 6.9 su
PLAT234_ALERT_4_C Large Hirshfeld Difference C11 -- C23 .. 0.24 Ang.
PLAT241_ALERT_2_C High Ueq as Compared to Neighbors for ..... S1 Check

And 2 other PLAT241 Alerts

PLAT241_ALERT_2_C High Ueq as Compared to Neighbors fc
PLAT241_ALERT_2_C High Ueq as Compared to Neighbors fc
PLAT242_ALERT_2_C Low Ueq as Compared to Neighbors for ..... Ru1 Check

Alert level G

PLAT002_ALERT_2_G Number of Distance or Angle Restraints on AtSite 4 Note

PLAT005_ALERT_5_G No _iucr_refine_instructions_details in the CIF Please Do !

PLAT007_ALERT_5_G Number of Unrefined Donor-H Atoms .... 3 Report

PLAT199_ALERT_1_G Reported _cell_measurement_temperature ..... (K) 293 Check

PLAT200_ALERT_1_G Reported _diffrn_ambient_temperature ..... (K) 293 Check

PLAT300_ALERT_4_G Atom Site Occupancy of *C12 is Constrained at 0.500 Check

And 19 other PLAT300 Alerts

PLAT300_ALERT_4_G Atom Site Occupancy of <C23 is Constra
PLAT300_ALERT_4_G Atom Site Occupancy of <C24 is Constra
PLAT300_ALERT_4_G Atom Site Occupancy of *H2 is Constra
PLAT300_ALERT_4_G Atom Site Occupancy of *H3 is Constra
PLAT300_ALERT_4_G Atom Site Occupancy of *H11A is Constra
PLAT300_ALERT_4_G Atom Site Occupancy of *H11B is Constra
PLAT300_ALERT_4_G Atom Site Occupancy of *H12A is Constra
PLAT300_ALERT_4_G Atom Site Occupancy of *H12B is Constra
PLAT300_ALERT_4_G Atom Site Occupancy of *H12C is Constra
PLAT300_ALERT_4_G Atom Site Occupancy of <H11C is Constra
PLAT300_ALERT_4_G Atom Site Occupancy of <H11D is Constra
PLAT300_ALERT_4_G Atom Site Occupancy of <H11E is Constra
PLAT300_ALERT_4_G Atom Site Occupancy of <H11F is Constra
PLAT300_ALERT_4_G Atom Site Occupancy of <H23A is Constra
PLAT300_ALERT_4_G Atom Site Occupancy of <H23B is Constra
PLAT300_ALERT_4_G Atom Site Occupancy of <H23C is Constra
PLAT300_ALERT_4_G Atom Site Occupancy of <H24A is Constra
PLAT300_ALERT_4_G Atom Site Occupancy of <H24B is Constra
PLAT300_ALERT_4_G Atom Site Occupancy of <H24C is Constra
PLAT301_ALERT_3_G Main Residue Disorder .... Percentage = 3 Note
PLAT720_ALERT_4_G Number of Unusual/Non-Standard Labels .... 3 Note
PLAT860_ALERT_3_G Number of Least-Squares Restraints .... 4 Note

0 **ALERT level A** = Most likely a serious problem - resolve or explain
1 **ALERT level B** = A potentially serious problem, consider carefully

8 **ALERT level C** = Check. Ensure it is not caused by an omission or oversight
28 **ALERT level G** = General information/check it is not something unexpected

2 ALERT type 1 CIF construction/syntax error, inconsistent or missing data
7 ALERT type 2 Indicator that the structure model may be wrong or deficient
3 ALERT type 3 Indicator that the structure quality may be low
23 ALERT type 4 Improvement, methodology, query or suggestion
2 ALERT type 5 Informative message, check

---

checkCIF publication errors

Alert level A

PUBL002_ALERT_1_A	The contact author's address is missing,
	_publ_contact_author_address.
PUBL005_ALERT_1_A	_publ_contact_author_email, _publ_contac
	_publ_contact_author_phone are all missing.
	At least one of these should be present.
PUBL006_ALERT_1_A	_publ_requested_journal is missing
	e.g. 'Acta Crystallographica Section C'
PUBL008_ALERT_1_A	_publ_section_title is missing. Title of
PUBL009_ALERT_1_A	_publ_author_name is missing. List of au
PUBL010_ALERT_1_A	_publ_author_address is missing. Author(
PUBL012_ALERT_1_A	_publ_section_abstract is missing.
	Abstract of paper in English.

---

7 **ALERT level A** = Data missing that is essential or data in wrong format
0 **ALERT level G** = General alerts. Data that may be required is missing

---

Publication of your CIF
You should attempt to resolve as many as possible of the alerts in all categories. Often the minor alerts point to easily fixed oversights, errors and omissions in your CIF or refinement strategy, so attention to these fine details can be worthwhile. In order to resolve some of the more serious problems it may be necessary to carry out additional measurements or structure refinements. However, the nature of your study may justify the reported deviations from journal submission requirements and the more serious of these should be commented upon in the discussion or experimental section of a paper or in the "special_details" fields of the CIF. *checkCIF* was carefully designed to identify outliers and unusual parameters, but every test has its

limitations and alerts that are not important in a particular case may appear. Conversely, the absence of alerts does not guarantee there are no aspects of the results needing attention. It is up to the individual to critically assess their own results and, if necessary, seek expert advice.
If level A alerts remain, which you believe to be justified deviations, and you intend to submit this CIF for publication in a journal, you should additionally insert an explanation in your CIF using the Validation Reply Form (VRF) below. This will allow your explanation to be considered as part of the review process.

Validation response form

Please find below a validation response form (VRF) that can be filled in and pasted into your CIF.

```
# start Validation Reply Form
_vrf_PUBL002_GLOBAL
;
PROBLEM: The contact author's address is missing,
RESPONSE: ...
;
_vrf_PUBL005_GLOBAL
;
PROBLEM: _publ_contact_author_email, _publ_contact_author_f
RESPONSE: ...
;
_vrf_PUBL006_GLOBAL
;
PROBLEM: _publ_requested_journal is missing
RESPONSE: ...
;
_vrf_PUBL008_GLOBAL
;
PROBLEM: _publ_section_title is missing. Title of paper.
RESPONSE: ...
;
_vrf_PUBL009_GLOBAL
;
PROBLEM: _publ_author_name is missing. List of author(s) na
RESPONSE: ...
;
_vrf_PUBL010_GLOBAL
;
PROBLEM: _publ_author_address is missing. Author(s) address
RESPONSE: ...
;
_vrf_PUBL012_GLOBAL
;
PROBLEM: _publ_section_abstract is missing.
RESPONSE: ...
```

;
end Validation Reply Form

If you wish to submit your CIF for publication in Acta Crystallographica Section C or E, you should upload your CIF via the web. If your CIF is to form part of a submission to another IUCr journal, you will be asked, either during electronic submission or by the Co-editor handling your paper, to upload your CIF via our web site.

PLATON version of 21/06/2015; check.def file version of 21/06/2015

Datablock I - ellipsoid plot

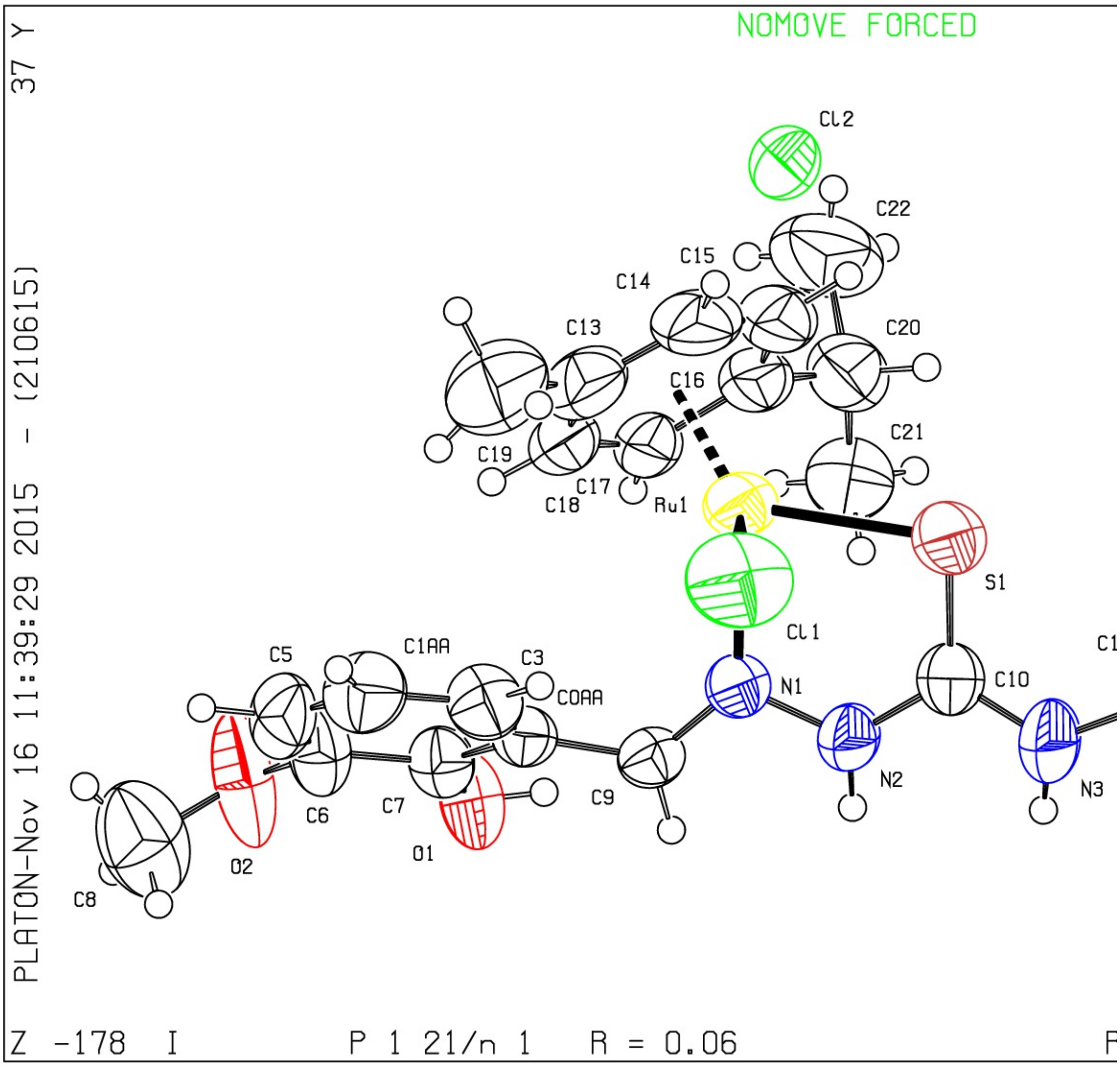

Download CIF editor (publCIF) from the IUCr
Download CIF editor (enCIFer) from the CCDC
Test a new CIF entry

checkCIF () running

Checking for embedded fcf data in CIF ...
No extractable fcf data in found in CIF

checkCIF/PLATON (full publication check)

You have not supplied any structure factors. As a result the full set of tests cannot be run.

THIS REPORT IS FOR GUIDANCE ONLY. IF USED AS PART OF A REVIEW PROCEDURE FOR PUBLICATION, IT SHOULD NOT REPLACE THE EXPERTISE OF AN EXPERIENCED CRYSTALLOGRAPHIC REFEREE.You have not supplied any structure factors. As a result the full set of tests cannot be run.

```
No syntax errors found.
Please wait while processing ....
```

CIF c
Inter

Datablock: I

Bond precision:	C-C = 0.0031 A		Wavelength=1.54184
Cell:	a=13.39352(8)	b=11.20615(8)	c=18.31082(11)
	alpha=90	beta=97.7958(6)	gamma=90
Temperature:	293 K		

	Calculated	Reported
Volume	2722.87(3)	2722.87(3)
Space group	P 21/c	P 1 21/c 1
Hall group	-P 2ybc	-P 2ybc
Moiety formula	C25 H29 Cl N3 O2 Ru S, Cl, H2 O	C25 H29 Cl N3 O2 Ru S, Cl, H2 O
Sum formula	C25 H31 Cl2 N3 O3 Ru S	C25 H31 Cl2 N3 O3 Ru S
Mr	625.56	625.56
Dx,g cm-3	1.526	1.526

Z	4	4
Mu (mm-1)	7.441	7.441
F000	1280.0	1280.0
F000'	1287.26	
h,k,lmax	16,13,22	16,13,22
Nref	5270	5223
Tmin,Tmax	0.394,0.640	0.680,1.000
Tmin'	0.285	

Correction method= # Reported T Limits: Tmin=0.680 Tmax=1.000 AbsCorr = MULTI-SCAN

Data completeness= 0.991 Theta(max)= 70.943

R(reflections)= 0.0203(4987) wR2(reflections)= 0.0531(5223)

S = 1.042 Npar= 321

The following ALERTS were generated. Each ALERT has the for
test-name_ALERT_alert-type_alert-level.
Click on the hyperlinks for more details of the test.

Alert level G

PLAT005_ALERT_5_G No _iucr_refine_instructions_details in the CIF Please Do !

PLAT007_ALERT_5_G Number of Unrefined Donor-H Atoms .... 5 Report

PLAT142_ALERT_4_G su on b - Axis Small or Missing .... 0.00008 Ang.

PLAT143_ALERT_4_G su on c - Axis Small or Missing .... 0.00011 Ang.

PLAT199_ALERT_1_G Reported _cell_measurement_temperature ..... (K) 293 Check

PLAT200_ALERT_1_G Reported _diffrn_ambient_temperature ..... (K) 293 Check

PLAT232_ALERT_2_G Hirshfeld Test Diff (M-X) Ru1 -- S1 .. 6.5 su

---

0 **ALERT level A** = Most likely a serious problem - resolve or explain
0 **ALERT level B** = A potentially serious problem, consider carefully
0 **ALERT level C** = Check. Ensure it is not caused by an omission or oversight

7 **ALERT level G** = General information/check it is not something unexpected

2 ALERT type 1 CIF construction/syntax error, inconsistent or missing data
1 ALERT type 2 Indicator that the structure model may be wrong or deficient
0 ALERT type 3 Indicator that the structure quality may be low
2 ALERT type 4 Improvement, methodology, query or suggestion
2 ALERT type 5 Informative message, check

checkCIF publication errors

Alert level A

PUBL002_ALERT_1_A The contact author's address is missing, _publ_contact_author_address.
PUBL005_ALERT_1_A _publ_contact_author_email, _publ_contac
_publ_contact_author_phone are all missing.
At least one of these should be present.
PUBL006_ALERT_1_A _publ_requested_journal is missing
e.g. 'Acta Crystallographica Section C'
PUBL008_ALERT_1_A _publ_section_title is missing. Title of
PUBL009_ALERT_1_A _publ_author_name is missing. List of au
PUBL010_ALERT_1_A _publ_author_address is missing. Author
PUBL012_ALERT_1_A _publ_section_abstract is missing.
Abstract of paper in English.

7 **ALERT level A** = Data missing that is essential or data in wrong format
0 **ALERT level G** = General alerts. Data that may be required is missing

Publication of your CIF
You should attempt to resolve as many as possible of the alerts in all categories. Often the minor alerts point to easily fixed oversights, errors and omissions in your CIF or refinement strategy, so attention to these fine details can be worthwhile. In order to resolve some of the more serious problems it may be necessary to carry out additional measurements or structure refinements. However, the nature of your study may justify the reported deviations from journal submission requirements and the more serious of these should be commented upon in the discussion or experimental section of a paper or in the "special_details" fields of the CIF. *checkCIF* was carefully designed to identify outliers and unusual parameters, but every test has its limitations and alerts that are not important in a particular case may appear. Conversely, the absence of alerts does not guarantee there are no aspects of the results needing attention. It is up to the individual to critically assess their

own results and, if necessary, seek expert advice.
If level A alerts remain, which you believe to be justified deviations, and you intend to submit this CIF for publication in a journal, you should additionally insert an explanation in your CIF using the Validation Reply Form (VRF) below. This will allow your explanation to be considered as part of the review process.

Validation response form

Please find below a validation response form (VRF) that can be filled in and pasted into your CIF.

```
# start Validation Reply Form
_vrf_PUBL002_GLOBAL
;
PROBLEM: The contact author's address is missing,
RESPONSE: ...
;
_vrf_PUBL005_GLOBAL
;
PROBLEM: _publ_contact_author_email, _publ_contact_author_f
RESPONSE: ...
;
_vrf_PUBL006_GLOBAL
;
PROBLEM: _publ_requested_journal is missing
RESPONSE: ...
;
_vrf_PUBL008_GLOBAL
;
PROBLEM: _publ_section_title is missing. Title of paper.
RESPONSE: ...
;
_vrf_PUBL009_GLOBAL
;
PROBLEM: _publ_author_name is missing. List of author(s) na
RESPONSE: ...
;
_vrf_PUBL010_GLOBAL
;
PROBLEM: _publ_author_address is missing. Author(s) address
RESPONSE: ...
;
_vrf_PUBL012_GLOBAL
;
PROBLEM: _publ_section_abstract is missing.
RESPONSE: ...
;
# end Validation Reply Form
```

If you wish to submit your CIF for publication in Acta Crystallographica Section C or E, you should upload your CIF via the web. If your CIF is to form part of a submission to another IUCr journal, you will be asked, either during electronic submission or by the Co-editor handling your paper, to upload your CIF via our web site.

PLATON version of 21/06/2015; check.def file version of 21/06/2015

Datablock I - ellipsoid plot

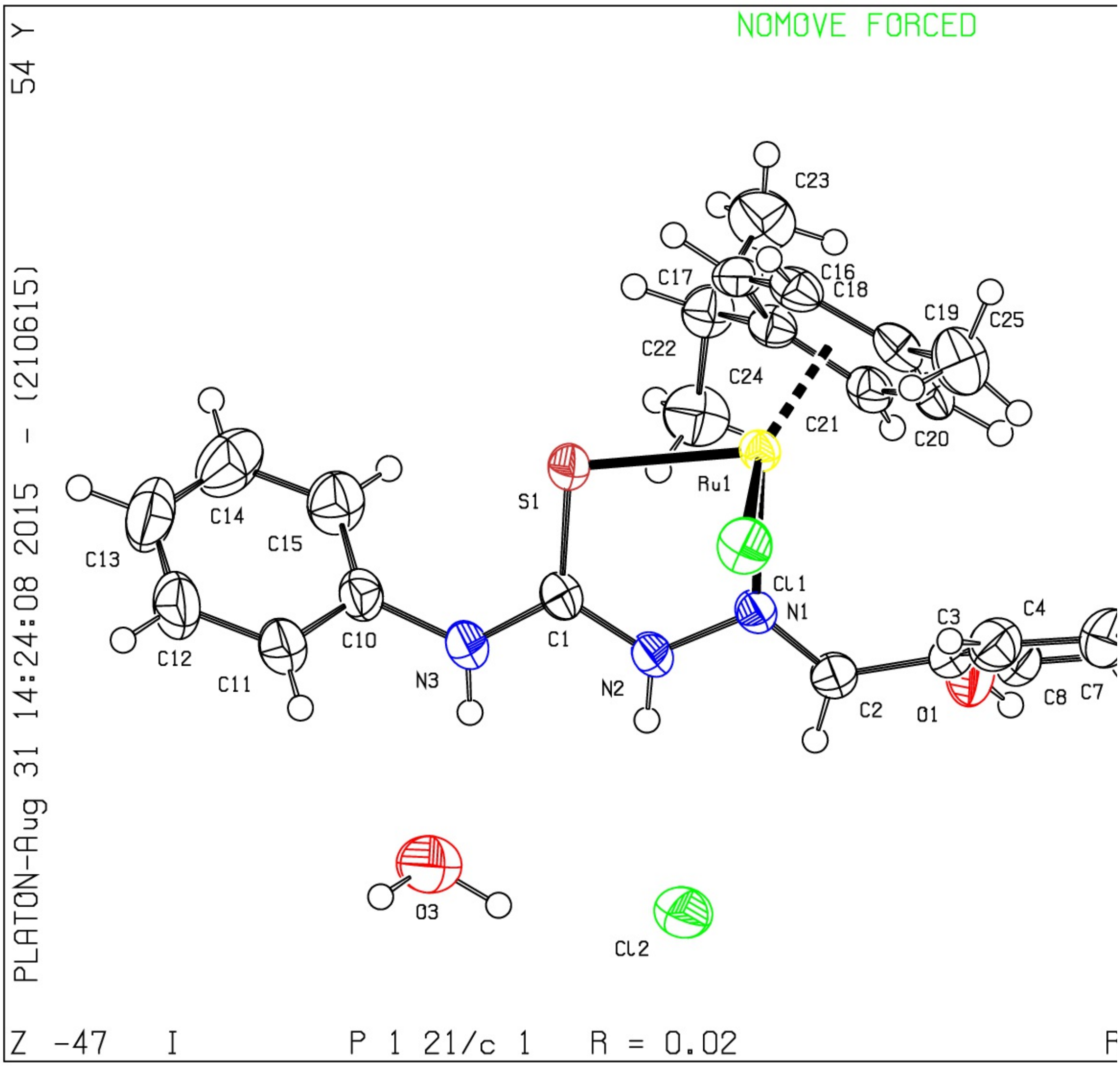

Download CIF editor (publCIF) from the IUCr
Download CIF editor (enCIFer) from the CCDC
Test a new CIF entry
